# Structure–function analysis of pectate lyase Pel3 reveals essential facets of protein recognition by the bacterial type 2 secretion system

**DOI:** 10.1016/j.jbc.2021.100305

**Published:** 2021-01-16

**Authors:** Camille Pineau, Natalia Guschinskaya, Isabelle R. Gonçalves, Florence Ruaudel, Xavier Robert, Patrice Gouet, Lionel Ballut, Vladimir E. Shevchik

**Affiliations:** 1 Microbiologie Adaptation et Pathogénie, Univ Lyon, Université de Lyon 1, UMR5240 CNRS, Villeurbanne, France; 2 Microbiologie Adaptation et Pathogénie, Univ Lyon, INSA Lyon, UMR5240, Villeurbanne, France; 3 Molecular Microbiology and Structural Biochemistry, Univ Lyon, UMR5086 CNRS, Lyon, France

**Keywords:** type II secretion system (T2SS), protein secretion, bacterial pathogenesis, protein structure, structure–function, virulence factor, pectinase, plant pathogen, *Dickeya*, *Pectobacterium*, CD, catalytic domain, GSP, general secretory pathway, IPTG, isopropyl-β-d-thiogalactopyranoside, Pel, pectate lyase, PL-3, polysaccharide lyases of family 3, r.m.s.d., root-mean-square deviation, T2SS, type 2 secretion system, T3SS, type 3 secretion system

## Abstract

The type II secretion system (T2SS) transports fully folded proteins of various functions and structures through the outer membrane of Gram-negative bacteria. The molecular mechanisms of substrate recruitment by T2SS remain elusive but a prevailing view is that the secretion determinants could be of a structural nature. The phytopathogenic γ-proteobacteria, *Pectobacterium carotovorum* and *Dickeya dadantii*, secrete similar sets of homologous plant cell wall degrading enzymes, mainly pectinases, by similar T2SSs, called Out. However, the orthologous pectate lyases Pel3 and PelI from these bacteria, which share 67% of sequence identity, are not secreted by the counterpart T2SS of each bacterium, indicating a fine-tuned control of protein recruitment. To identify the related secretion determinants, we first performed a structural characterization and comparison of Pel3 with PelI using X-ray crystallography. Then, to assess the biological relevance of the observed structural variations, we conducted a loop-substitution analysis of Pel3 combined with secretion assays. We showed that there is not one element with a definite secondary structure but several distant and structurally flexible loop regions that are essential for the secretion of Pel3 and that these loop regions act together as a composite secretion signal. Interestingly, depending on the crystal contacts, one of these key secretion determinants undergoes disorder-to-order transitions that could reflect its transient structuration upon the contact with the appropriate T2SS components. We hypothesize that such T2SS-induced structuration of some intrinsically disordered zones of secretion substrates could be part of the recruitment mechanism used by T2SS.

The Gram-negative bacteria possess a multilayer cell envelope composed of the inner membrane surrounding the cytoplasm and the outer membrane facing the external medium. The two membranes delimit an extracytoplasmic compartment, the periplasm, which contains a peptidoglycan layer (1, 2). To ensure the selective transport of proteins and other macromolecules across this complex cell envelope, bacteria have evolved several specialized cell machineries (3, 4, 5, 6). The type II secretion system (T2SS) is widespread among Gram-negative bacteria, and it is used to secrete fully folded proteins, usually lytic enzymes and toxins, from the periplasm into the external medium or host tissue (7, 8, 9, 10). For instance, the phytopathogenic γ-proteobacteria, *Pectobacterium carotovorum* and *Dickeya dadantii*, cause soft rot disease in a variety of plants through the action of several pectinases secreted by the T2SS, called Out (11, 12). The T2SS is a sophisticated transenvelope scaffold that is composed of at least 12 conserved core elements, generically called GspC to GspO (for General Secretory Pathway) or, more specifically, OutC to OutO for *Pectobacterium* and *Dickeya*. An inner membrane platform, formed by GspC, L, M, and F, interacts with the cytoplasmic ATPase GspE. GspE is thought to energize the assembly of the periplasmic pseudopilus, composed of GspG, H, I, J, and K, which participates in the translocation of folded exoproteins through the proteinaceous channel formed by the outer membrane secretin GspD (8, 9, 10).

The folded nature of proteins secreted by T2SS, together with an apparent absence of any common linear sequences, led to a widely held hypothesis that the T2SS secretion determinants could be of a structural nature (13, 14, 15, 16). The T2SS is very versatile, and depending on the bacteria, it allows the secretion of up to 20 sequence-unrelated and structurally dissimilar proteins (11, 17, 18, 19). On the other hand, the same T2SS restricts the secretion of very similar orthologous proteins from other species. For example, the T2SS of *D. dadantii* secretes more than 15 different proteins, but it can discriminate between its own pectate lyase PelI and the Pel3 from *P. carotovorum*, although they share 67% of sequence identity (20). Recently, we have exploited this species-specific secretion to study the molecular mechanisms of substrate recognition by T2SS (21).

We have shown that PelI interacts with two T2SS components, the inner membrane GspC and the outer membrane GspD (21). In addition, we have found that an exposed 9 residue-long region, loop 3 of PelI, acts as a specific secretion signal that controls protein recruitment by the T2SS. The interaction of this loop with the dedicated domains of GspC and GspD is essential for the T2SS to discriminate between the cognate substrate PelI and heterologous Pel3. Furthermore, these data suggest that some other zones of PelI could also be involved in protein recruitment by the T2SS, indicating that this process is more complex than simply the recognition of a single loop (21).

Previous studies have largely benefited from the high-resolution structures available for the pectate lyase PelI and for the periplasmic domains of the GspC and GspD components (22, 23, 24, 25). However, the structure of Pel3 from *P. carotovorum* was still unresolved. It can be expected that the orthologous Pel3 and PelI would share a similar overall topology, but fine specific structural features may also exist since Pel3 is not recognized by the T2SS of *D. dadantii*. Therefore, we undertook a structural characterization of Pel3 to identify such potential secretion determinants and to carry out a rational design and construction of the Pel3 variants that could be secreted by *D. dadantii*.

We reveal that Pel3 shares the same general topology as PelI, consisting of an N-terminal domain of a fibronectin type III fold (Fn3) linked to a catalytic domain (CD) that adopts a parallel β-helix topology. Whereas the core structure of both Fn3 and CD is well conserved in Pel3 and PelI, significant differences were observed for several exposed loop regions, indicating that these are putative secretion determinants. To test this hypothesis, we systematically substituted such divergent zones of Pel3 with those from PelI and then assessed the secretion of the generated hybrids in *D. dadantii*. We have demonstrated that, in addition to loop 3 of Fn3, several other loop regions are essential for secretion. Some of them are spatially close to the loop 3 and could together constitute a composite secretion determinant, whereas others are more distant and may act as independent secretion signals. These data suggest that the proteins secreted by T2SS pass through a multifaceted control that monitors the adequacy of several secretion determinants. Remarkably, structural analysis of Pel3 reveals that the key secretion signal, loop 3 of Fn3, is present in the crystals in different conformations. Such conformational transitions could reflect a transient structuration of loop 3 when in contact with an appropriate T2SS component. We hypothesize that such T2SS-induced structuration of some intrinsically disordered zones of the protein to be secreted is part of the recruitment mechanism used by T2SS.

## Results

### Overall fold of the pectate lyase Pel3

**Figure 1:**
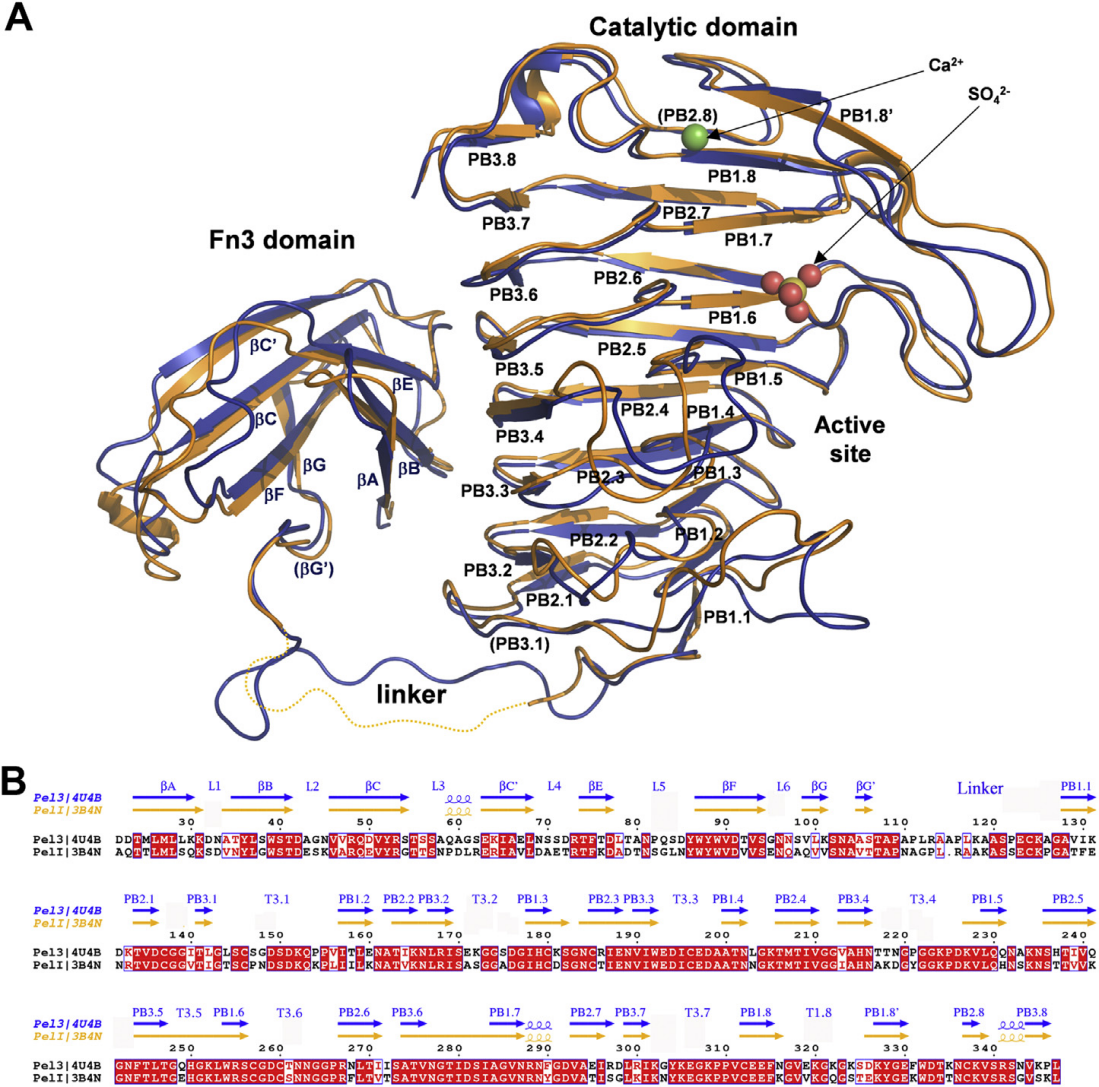
**Structure-sequence comparison of the pectate lyases Pel3 and PelI.***A*, superimposition of Pel3_1m_ (*blue*, PDB 4U4B) and PelI (*orange*, PDB 3B4N) structures in ribbon representation. Bound calcium and sulfate ions are shown for Pel3. The disordered interdomain linker is not visible in PelI structure and is depicted by a *dashed line*. *B*, structure-based sequence alignment of Pel3 and PelI with secondary structure elements. The identical and similar residues are shown on *red* background.

We crystallized the full-length Pel3 in two types of monoclinic crystals with one or two monomers in the asymmetric unit, named Pel3_1m_ and Pel3_2m_, and solved the structures at 1.8 and 2.1 Å resolution, respectively (Table S1). The structures are very similar, with the same fold and domain arrangement, as testified by an r.m.s. deviation of 1.6 Å on all Cα pairs. Pel3 adopts a compact pear-like overall shape composed of two unequal domains, a small fibronectin type III domain (Fn3) (residues 1–109) and a large catalytic domain that has a β-helix fold (residues 120–347) (Fig. 1). The two domains are linked by a decapeptide segment (residues 110–119), which is observed in the electron density map of Pel3_1m_, and have the same respective orientation in Pel3_1m_ and Pel3_2m_. When superimposed onto the structure of the orthologous PelI from *D. dadantii*, the same domain composition and overall shape are observed, with an r.m.s. deviation of 2 Å on all Cα pairs (Fig. 1*A*).

### Organization of the catalytic domain

**Figure 2:**
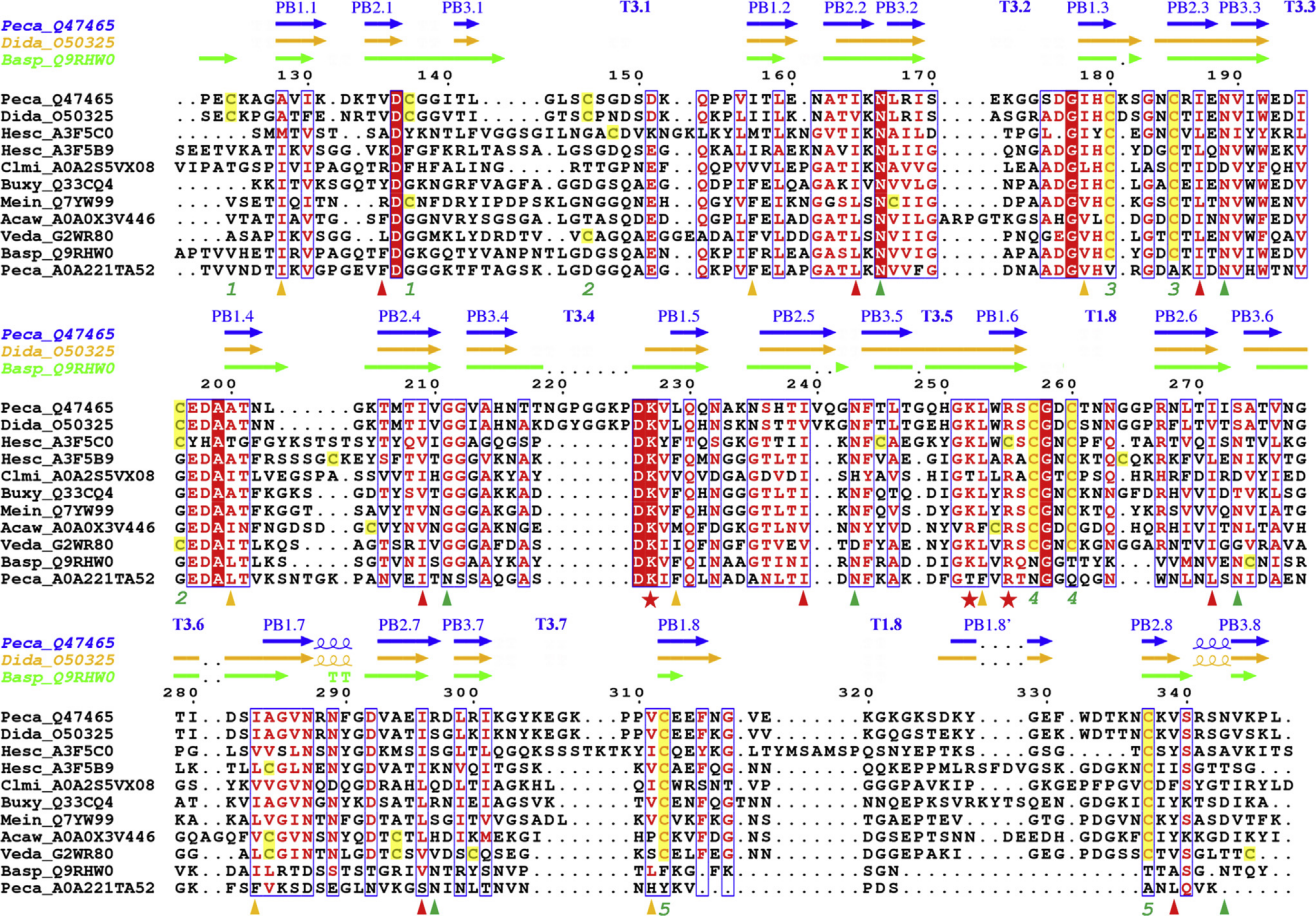
**Family sequence alignment of PL-3 representative examples**. Shown are: *P. carotovorum* Pel3 (Q47465); *D. dadantii* PelI (O50325); *Heterodera schachtii* Pel1 (A3F5C0); *H. schachtii* Pel2 (A3F5B9); *Clavibacter michiganensis* Pel (A0A2S5VX08); *Bursaphelenchus xylophilus* BxPel1 (Q33CQ4); *Meloidogyne incognita* Pel (Q7YW99); *Actinoplanes awajinensis* Pel (A0A0X3V446); *Verticillium dahliae* (G2WR80); *Bacillus sp.* KSM-P15 Pel (Q9RHW0) and *Pectobacterium carotovorum* HrpW (A0A221TA52), representative of various phylogenetic groups (Fig. 5). In brackets are the corresponding UniProtKB codes. The secondary structure elements are shown for *P. carotovorum* Pel3 (PDB 4U4B), *D. dadantii* PelI (PDB 3B4N), and *Bacillus sp.* KSM-P15 Pel (PDB 1EE6), in *blue*, *orange*, and *green*, respectively. The residue numbering is shown for Pel3. The residues conserved in the PL-3 family members are in *red*. The residues of two hydrophobic ladders and an Asn stacking are shown with the *orange*, *red*, and *green triangles*, respectively. *Red asterisks* indicate the catalytic residues. The cysteine residues are highlighted in *yellow* and the disulfide bonds of Pel3 are numbered from 1 to 5 with *green numbers*. The figure was generated with the ESPript server (57).

The pectate lyases (EC 4.2.2.2 and EC 4.2.2.9) catalyze the cleavage of polymeric α-1-4-linked polygalacturonic acid within the pectin component of the plant cell wall, leaving an unsaturated C_4_–C_5_ bond at the newly formed nonreducing end (24). Pectate lyases have been classified into five structurally and phylogenetically unrelated families of polysaccharide lyases (PL-1, 2, 3, 9, and 10) (www.cazy.org, (26)). The catalytic domain of Pel3 adopts the general β-helix fold typical of the structurally characterized proteins from the PL-3 family: it is made up of eight right-handed coils stacked on top of one another. Each coil consists of three consecutive strand-turn motifs, termed PBn.m-Tn.m, where n is 1 to 3 and m is 1 to 8 (Fig. 1). Five disulfide bonds are well conserved between PelI and Pel3 (Fig. S1): three of them (124/137, #1, 180/185, #3, and 312/337, #5) reinforce the stability of the β-solenoid fold while the two other disulfide bonds (146/196, #2 and 257/260, #4) fasten the positions of the extended loops. The hydrophobic core of the Pel3 β-helix is stabilized by a series of hydrophobic interactions between the inward-pointing side chains of several aliphatic residues, Ile, Val, and Leu. These residues are organized into two regular ladders, extending along the whole length of the β-helix, at the β-strands PB1 and PB3, respectively (Fig. S2). These aliphatic interstrand stackings are well conserved among the PL-3 family members, while in some proteins, Phe is present instead of aliphatic residues (Fig. 2).

The Pel3 catalytic site is nearly identical to that of PelI and similar to those of two other structurally characterized PL-3 members from *Bacillus* sp. KSM-P15 and from *Caldicellulosiruptor bescii* (PDB entries 1EE6 and 3T9G, respectively) (27, 28). It carries the same invariant residues with Lys227 acting as the catalytic base and Lys252 and Arg255 implicated in the binding of the substrate (Fig. 2 and Fig. S3). In the catalytic site of Pel3_1m_, a sulfate ion was detected, which forms salt bridges with Lys227, Lys252, and Arg255 mimicking the hydroxyl groups of the natural substrate, polygalacturonic acid (Fig. S4). In addition, a structural calcium ion was present in the monomer B of Pel3_2m_ where it coordinates the main-chain carbonyl O of Ile195 and Pro221, the side-chain Oδ atom of Asp194 and Asn216, and two water molecules, which complete the pentagonal bipyramidal geometry (Fig. S3*A*). Superimposition of monomers A and B of Pel3_2m_ indicated that the Ca^2+^ ion seems to be necessary for a proper fold of loop T3.4. Indeed, in the monomer B, T3.4 forms a lid over the bound Ca^2+^, whereas this turn is highly destabilized, in the monomer A, in the absence of calcium (Fig. S5). Interestingly, this arrangement is specific to Pel3 and PelI as the loop forming T3.4 is absent from other pectate lyases of the PL-3 family (Fig. 2 and Fig. S3).

**Figure 3:**
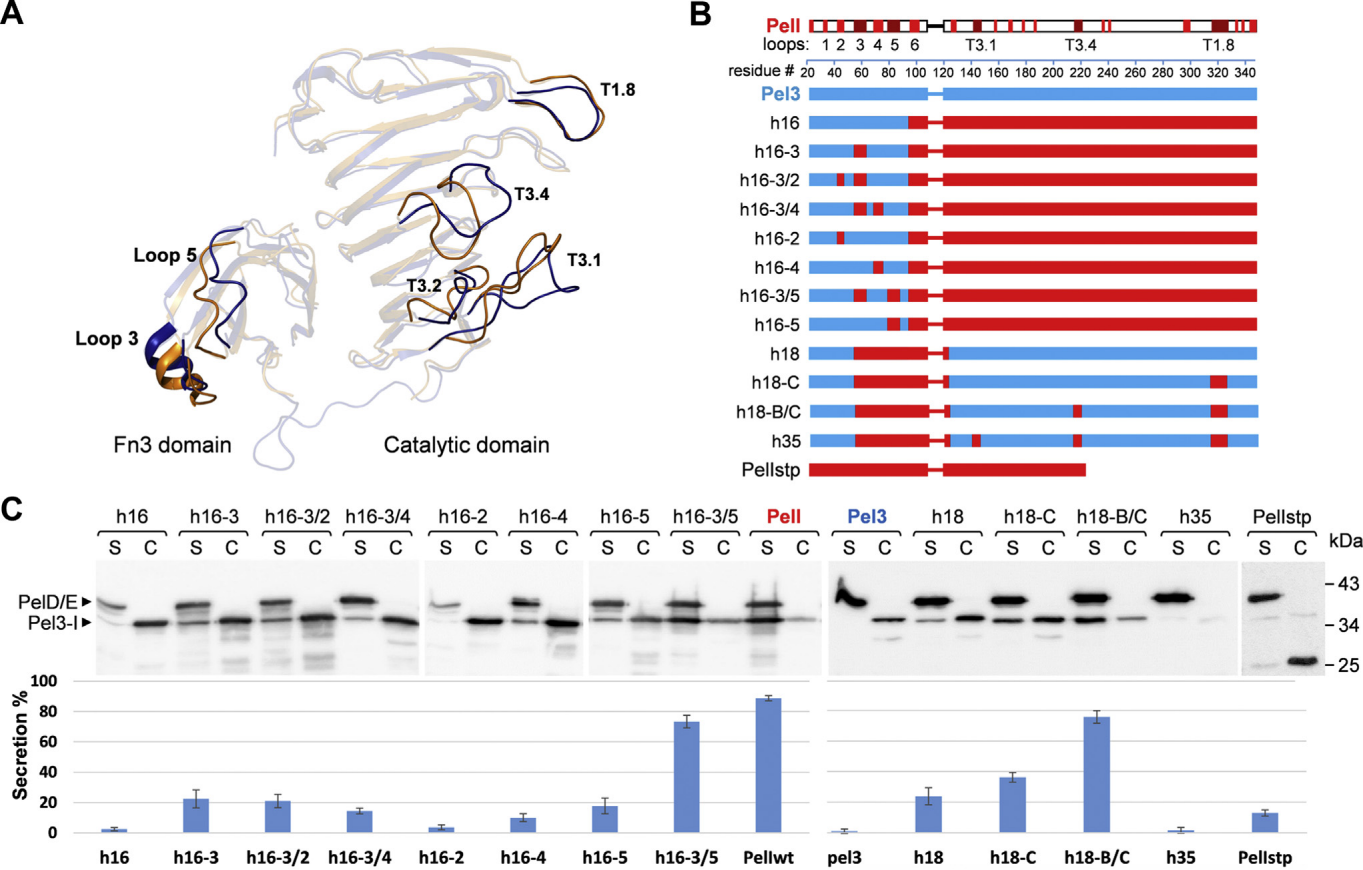
**Loop substitution analysis between Pel3 and PelI reveals several secretion-relevant protein zones.***A*, structural variations between Pel3 and PelI loop regions: superimposition of Pel3 (*blue*) and PelI (*orange*) in ribbon representation shown in transparency. Loops T1.8, T3.1, T3.2, and T3.4 from the catalytic domain and loops 3 and 5 from the Fn3 domain used for chimeric Pel3/PelI constructs are shown in solid mode. The superimposition is made by using only the catalytic domains as templates. *B*, schematics of the used Pel3/PelI hybrids and their secretion efficiencies: zones from Pel3 are in *blue* and those from PelI are in *red*. The precise amino acid sequences are shown on Fig. S10. *C*, secretion assays with the Pel3/PelI hybrids. The Pel3/PelI variants shown in panel *B* were expressed from a plasmid in *D. dadantii* A5159 *pelI*. Then, culture supernatants (S) and cells (C) were separated on SDS-PAGE and analyzed by immunoblotting with antibodies raised against pectate lyases PelI and PelD. The chromosome-expressed PelD is used as a positive secretion control to verify if secretion of other T2SS substrates is not affected by the expression of Pel3/PelI hybrids. The histogram shows the percentage of Pel3/PelI hybrids secreted in the culture supernatant. Each bar shows the percentage of secreted hybrid relative to the whole amount of the corresponding hybrid detected in culture supernatant and cells, S/(S + C). The bars are positioned below the corresponding gel lines. The bars represent the mean values ± SD of three independent experiments.

Superimposition of the catalytic domains of Pel3 and PelI reveals some subtle structural differences that are essentially restricted to the extended loops formed by T3.1, T3.2, T3.4, and T1.8 (Fig. 3*A*). These zones have a low degree of sequence conservation and show variable arrangements in Pel3 and PelI. Notably, these loops are also differently arranged in the various crystal forms of Pel3, indicating their high intrinsic flexibility (Fig. S6). Such properties could be consistent with the formation of specific secretion patterns for cognate T2SS.

### Fn3 domain

The N-terminal domain of Pel3 has a seven-stranded Fn3 fold very similar to that of the Fn3 domain of PelI, with an r.m.s. deviation of 1.0 Å on all Cα pairs. It is composed of two antiparallel β-sheets packed against each other (Fig. 1). Except for Pel3, PelI, and a few close homologs from γ-proteobacteria, other characterized representatives of the PL-3 family do not possess an Fn3-like domain. Some PL-3 members carry, instead, a carbohydrate-binding module (Firmicutes and Fungi) or a ricin B-like lectin domain (Actinobacteria), while the Nematode’s pectate lyases usually consist of the catalytic PL-3 domain only (Table S2). The Fn3 domain does not affect the catalytic activity of PelI and its biological function remains unclear (24). Fn3-like modules have also been identified in some other carbohydrate-active enzymes suggesting its possible implication in plant cell wall degradation (29, 30, 31).

The Fn3 topology is, however, very common in eukaryotes, and it has been found in about 2% of all animal proteins (32). Consistent with these observations, a search for structural homologs of Fn3 Pel3, using the Dali server (33), shows that the highest scoring hits are the Fn3 domains from eukaryotic proteins (Fig. S7 and Table S3). In contrast, a few characterized Fn3 domains from bacterial carbohydrate-active enzymes are structurally divergent from Fn3 Pel3. Regardless of the level of structural similarity of the considered Fn3 domains, their sequence identity is low (less than 15% for the best hits) and only a few aromatic and/or hydrophobic core-forming residues are conserved across these Fn3 domains (Fig. S7). In addition, the loop/turn regions vary widely, even between the orthologous Fn3 domains of Pel3 and PelI where the sequence identity is very poor (Fig. 1*B* and Fig. S7). This could be consistent with the specific functions of these zones. For instance, in some eukaryotic Fn3 domains, loop regions have been shown to constitute binding sites for cognate protein partners (34, 35, 36).

### Varied structuration of the key secretion signal, loop 3 of Fn3 Pel3

**Figure 4:**
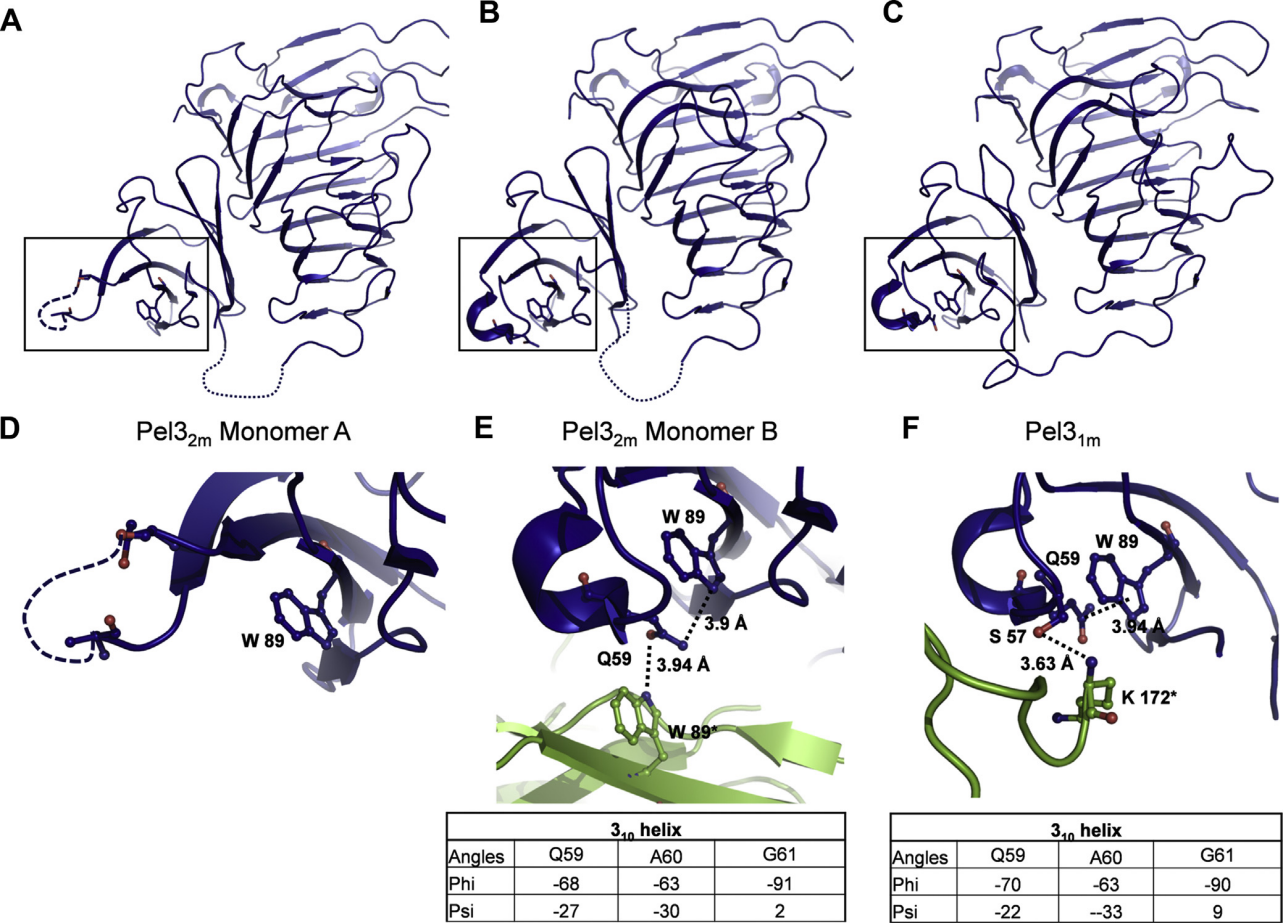
**Variable structural organization of loop 3 Fn3 and interdomain linker in Pel3.***A*–*C*, organization of the loop 3 Fn3 observed respectively, in Pel3_2m_ (PDB 4U49) monomer A (*A*) and monomer B (*B*) and Pel3_1m_ (PDB 4U4B) (*C*). *D*–*F*, close-up view of the protein zones surrounded in *A*–*C*, respectively, showing crystal contacts with neighboring Pel3 monomers (in *green*) as well as phi and psi angles of residues Gln59, Ala60, and Gly61 forming a 3_10_-helix of Pel3 loop 3.

Comparison of the three monomer structures of Pel3 observed in the different crystals shows that loop 3 of Fn3, located between strands βC and βC’, adopts distinct conformations depending on the intra- and intermolecular contacts. Notably, in the Pel3_2m_ monomer A, no electronic density corresponding to loop 3 could be detected, indicating that it is completely disordered (Fig. 4*A*). However, monomer B presents a well-structured loop with a 3_10_-helix (Fig. 4*B*). The helix seems to be stabilized by Q59, which is sandwiched between two tryptophan residues, W89 from the same monomer B and W89∗ from monomer A. A similar conformation with a 3_10_-helix is observed in Pel3_1m_, where loop 3 is stabilized, by two hydrogen bonds, with another monomer and by a polar-π interaction between Q59 and W89 in the same monomer (Fig. 4*C* and Fig. S8*B*).

Interestingly, a similar cation–π interaction is observed in the crystal of PelI, where R60 interacts with W87 and the loop 3 Fn3 is neatly ordered in a 3_10_-helix (Fig. S8*A*). In PelI, the 3_10_-helix is additionally stabilized by a salt bridge between D58 and K328∗, as well by a hydrogen bond between N56 and Y325∗. Since the loop 3 of Fn3 has been shown to interact with the cognate T2SS (21), it is tempting to hypothesize that such transient interactions with the T2SS components could play a similar structuring role during the Pel3/PelI recruitment by the secretion system.

### Fn3/CD interdomain interface

The two domains of Pel3 form a closed structure with a buried surface area of 880 Å^2^ stabilized by a series of hydrogen bonds and ionic interactions (Fig. S9). The Fn3 side of the interdomain interface includes several residues of strands βA, βB, and βE. On the CD side of the interface, the residues Asn166, Asn189, and Asn243 form a “Velcro”-like motif that offers a series of ionic and polar groups stabilizing the Fn3/CD interface. These residues form the turns T2 at coils 2, 3, and 5 of the β-helix, and they are well conserved among the PL-3 family members as a part of an Asn stacking (Fig. 2 and Fig. S2). In Pel3 and PelI, the Fn3 domain covers these residues of the CD until the protein is secreted from the periplasm by the T2SS. Once secreted *in planta*, the Fn3 domain of Pel3/PelI is cleaved off from the catalytic domain by the bacterial proteases (37).

A similar compact organization is observed for PelI, with an interdomain buried contact surface of 750 Å^2^ (Fig. S9). The interdomain interface has a similar orientation in Pel3 and PelI and most interacting residue pairs are conserved, though some specific contacts are more prominent in PelI. For instance, a salt bridge K29/E190 is present in the PelI interface but absent in Pel3 (Fig. S9). These subtle differences in organization of the interface cause some displacements of the Fn3 and CD domains relative to each other, in the Pel3 and PelI core structures. Indeed, when superimposing the catalytic domains of Pel3 and PelI, their respective Fn3 domains and, more specifically, the loops 3 and 5 are obviously shifted (Fig. 3*A*). Since the loop 3 of Fn3 has been previously identified as a key secretion signal, these differences could, in turn, affect recognition of the protein by the T2SS.

### Substitution analysis of the Fn3 domain

Comparison of Pel3 and PelI revealed that their core structures composed of β-strands are highly conserved, whereas the arrangement of several extended loop regions of both Fn3 and CD varies significantly (Fig. 3*A*). To examine whether these regions could act as specific secretion signals, first, we systematically substituted these zones of Fn3 Pel3 with those from PelI and then assessed secretion of the generated hybrids in *D. dadantii*. As was expected from the previous study (21), the h16 hybrid, carrying the Fn3 of Pel3 and the CD of PelI, was not secreted by *D. dadantii* (Fig. 3, *B* and *C*). Substitution of the loop 3 slowly improved secretion of the resulting hybrid h16-3 (Fig. 3, *B* and *C*), indicating the importance of this loop but suggesting that some other zones of Fn3 are also necessary for secretion. Additional substitutions of loop 2 and 4 did not have any visible effect (h16-3/2 and h16-3/4) but the introduction of the loop 5 from PelI significantly enhanced secretion of the resulting hybrid h16-3/5 (Fig. 3, *B* and *C*). Since the substitution of loop 5 alone (h16-5) improved secretion at a similar moderate level as with h16-3, the addition of these two substitutions in h16-3/5 has a clear synergistic effect. The loops 3 and 5 are adjacent in the Fn3 structure and could constitute a continuous binding interface, acting as a joint secretion determinant.

### Substitution analysis of the catalytic domain

To identify potential secretion determinants located at the catalytic domain, a similar loop-substitution strategy was employed. Hybrid h18, carrying a secretion-relevant portion of Fn3 PelI and the CD from Pel3, was barely secreted by *D. dadantii*, which corroborates the presence of specific secretion signals within the CD. To search for such presumed secretion determinants, h18 was used as a framework to introduce the selected zones from the PelI CD (Fig. 3, *B* and *C*). Structural comparison of Pel3 and PelI shows several extended loop regions of CD with variable arrangements and sequences, namely T3.1, T3.2, T3.4, and T1.8 (Fig. 3*A* and Fig. S6). The substitution of loop T1.8 obviously improved secretion of the resulting hybrid h18-C (Fig. 3, *B* and *C*, compare h18-C and h18). The subsequent introduction of T3.4 from PelI further enhanced secretion of h18-B/C (Fig. 3, *B* and *C*, compare h18-B/C with h18-C), indicating the importance of these two loops for protein secretion. On the other hand, the substitution of the turn T3.1 led to the degradation of the resulting hybrid h35 (Fig. 3, *B* and *C*). It seems that insertion of heterologous turn T3.1 could provoke some steric hindrances, thus destabilizing the overall protein fold. Collectively, these data show that two elements of the CD, T3.4 and T1.8, could constitute two additional species-specific secretion signals of Pel3/PelI. Furthermore, since these loops are located close enough to each other (Fig. 3*A*), it is possible that they form a common binding interface with an appropriate T2SS component.

### Role of the Fn3/CD interdomain interface

The secretion signals identified herein, notably the loops 3 and T1.8, are located at diametrically opposite sides of the Pel3/PelI core structure, respectively, at the Fn3 and the catalytic domain. These two domains interact *via* a large interdomain interface, which might still disassemble during the course of secretion. In this case, the secretion signals located at the Fn3 and the CD would be displaced with respect to each other. To test this hypothesis, the Fn3/CD interdomain interface of PelI was locked by an artificial disulfide bond. The resulting PelI variant, carrying the double cysteine substitution, T70C/N240C, was efficiently secreted (Fig. S11). Since disulfide bonds are formed in the periplasm prior to protein recruitment by the T2SS (38), this indicates that such a permanently locked PelI variant is very compatible with the T2SS. It seems plausible, therefore, that during secretion of the native protein, Fn3 and CD could also remain bound together as in the crystal structure. In this way, the reciprocal positioning of various secretion signals with respect to one another (*e.g.*, loop 3 of Fn3 and loop T1.8 of CD) would be preserved during protein recruitment by the T2SS.

To determine whether the simultaneous presence of both sets of secretion signals, from Fn3 and CD, is essential for Pel3/PelI secretion, we constructed a truncated PelI, PelIstp, lacking the C-terminal half of the catalytic domain and the related loop T1.8 (Fig. 3). This derivative was not secreted suggesting that protein recruitment by T2SS necessitates multisite recognition from the Fn3 and CD domains.

### Pel3 phylogeny

**Figure 5:**
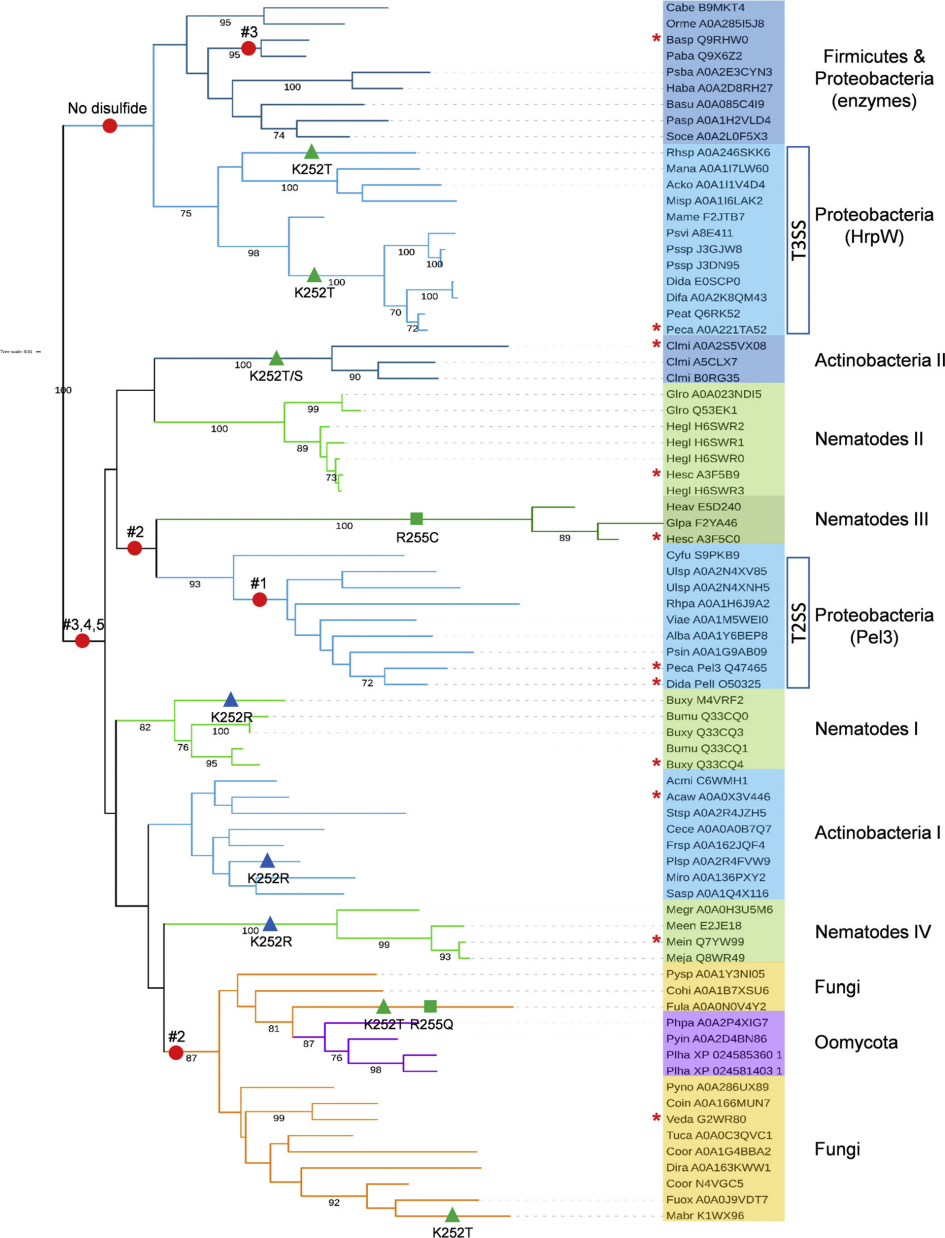
**Phylogenetic tree of Pel3 and the PL-3 family proteins.** Phylogenetic tree topology was obtained by using bootstrapped maximum-likelihood approach with PhyML (55) as described in “Experimental procedures” and visualized with iTOL software (56). Bootstrap values ≥70 are indicated below the branches. Colored branches and boxes show the PL-3 protein groups from Bacteria (*blue*), Nematodes (*green*), Fungi (*orange*), and Oomycetes (*magenta*). *Red circles* show the occurrence of disulfide bonds, numbered from #1 to 5, as in Pel3. *Green* and *blue triangles* indicate the substitutions of substrate-binding residues equivalent to K252T/S and K252R of Pel3, respectively. Full species names and some other features of the proteins used are listed in the Table S2. The sequences used for family sequence alignment (Fig. 2) are indicated with the *red stars*.

The catalytic domain of Pel3 belongs to the PL-3 family of polysaccharide lyases found in various groups of bacteria, fungi, oomycetes, and plant-associated nematodes (www.cazy.org, (26)). The bacterial PL-3 enzymes do not form a monophyletic clade but are interspersed between four distant groups (Fig. 5). *Pectobacterium*, *Dickeya*, and some other plant-pathogenic Proteobacteria possess two phylogenetically distant classes of PL-3, a Pel3/PelI orthologous group and HrpW-like proteins. HrpW is a T3SS component that binds the pectin and facilitates the passage of the T3SS needle through the plant cell wall (39, 40). The proteobacterial HrpWs carry a threonine in place of the residue equivalent to the K252 of Pel3 (Fig. 5). Interestingly, the substitutions equivalent to K252T/S/R and R255Q/C have occurred independently in several PL-3 groups (Fig. 5 and Table S2). The residues equivalent to K252 and R255 of Pel3 are involved in the substrate binding (Fig. S4) and their substitutions could allow for a better adaptation to a particular plant cell wall composition. For instance, the substitution equivalent to K252R permits enzymatic activity at a high concentration of calcium, conditions specific to the middle lamella (24).

HrpW proteins form a large cluster together with the group of PL-3 enzymes originating from Firmicutes and some Proteobacteria (Fig. 5). These proteins have no, or very occasionally one, disulfide bond, equivalent to #3 of Pel3. In contrast, all the other PL-3 groups possess the disulfide bonds equivalent to #3, 4, and 5 of Pel3 (Fig. 5 and Table S2). It seems likely that the HrpW/Firmicutes cluster was separated from the other groups before these disulfide bonds evolved in ancestral PL-3 proteins. The proteins of the HrpW/Firmicutes cluster carry a well-ordered, regular β-helix that is further stabilized by an Asn ladder extending along the whole length of the β-helix (Fig. S2). Generally, the interstrand Asn stacking acts as a glue, sticking together neighboring strands, both in parallel β-helices and in amyloid fibrils (41). Only a part of this Asn ladder is conserved in Pel3 and in many other PL-3 members (Fig. 2 and Fig. S2). Furthermore, the seventh and eighth coils of these proteins are significantly deformed by the presence of some additional loops, helices, and strands (Fig. S2). In these proteins, the disulfide bonds #3 and #5 act as a sort of a molecular clasp, reinforcing the basic β-helix framework, while #4 fastens the loop T1.6 that faces the catalytic groove and carries substrate-binding residues (24). Interestingly, the disulfide bond equivalent to #2 of Pel3 seems to have appeared independently in two distant groups, Fungi/Oomycetes and Proteobacterial PelI/Pel3, respectively (Fig. 5). This might corroborate the importance of this bond for the stabilization of protein structure (Fig. S5).

The disulfide bond equivalent to #1 of Pel3 (C124-C136) is only present in Pel3, PelI, and a few very close orthologs from γ-proteobacteria, all secreted by the T2SS. These pectate lyases are preceded by an N-terminal Fn3 domain, another exclusive feature of the Pel3/PelI group. The disulfide bond #1 attaches the beginning of the β-solenoid to the interdomain linker, thus maintaining the correct orientation of the Fn3 domain, which is essential for secretion. Finally, another secretion determinant, the extended loop T3.4, is also an exclusive feature of Pel3/PelI-like pectate lyases from γ-proteobacteria carrying a T2SS (Figs. 2 and 4). These data suggest a possible coevolution of Pel3/PelI orthologs for secretion by the T2SS.

## Discussion

In order to establish the specific determinants of the type 2 secretion, we performed a structural characterization of the pectate lyase Pel3 from *P. carotovorum* and compared it with its orthologous counterpart, PelI from *D. dadantii*. Then, to assess the biological relevance of the observed structural variations, we carried out a loop-substitution analysis combined with secretion assays.

The loop substitution, or grafting, approach is largely employed with the Fn3 scaffold to engineer artificial nonantibody binders of varying specificity (42, 43). To generate the binding interface of interest, the loops of selected binding specificity are grafted in place of the native loops into a basic Fn3 framework. We showed the adequacy of this technique with the Fn3 Pel3 domain, in which the two neighboring loops 3 and 5 have been identified as constituting a *bona fide* secretion signal. This approach also allowed us to identify two loop regions of the catalytic domain (T1.8 and T3.4) acting as another specific secretion determinant(s). Collectively, these data argue for a multisite recognition of Pel3/PelI by the T2SS. In agreement with this proposal, a truncated PelI derivative, lacking the C-terminal half of CD and carrying a reduced set of secretion signals, was not secreted. Some previous studies have also suggested that T2SS substrates possess two or more distantly located secretion signals (13, 15, 44). In this study, only the elements divergent between the two homologous proteins, Pel3 and PelI, were examined. Therefore, it could not be excluded that some other structural features, such as overall folding or prevalence of certain secondary elements (*e.g.*, beta-strands) could be important or directly implicated in substrate recruitment by T2SS.

Remarkably, loops 3 of Fn3 and T1.8 of CD are very distant from one another and located on the diametrically opposite sides of the Pel3 (Fig. 3*A*). Apparently, they could not constitute a continuous binding interface with only one domain of the dedicated T2SS component. Instead, they may be recognized through a coordinated interaction with at least two T2SS components, or domains, in a pincer-like mode. Alternatively, various secretion signals could interact successively with appropriate T2SS components during the course of secretion. In the latter case, the positioning of these signals relative to each other would probably vary. Indeed, it seems possible that during the protein recruitment and passage through the T2SS, the Fn3 and CD domains could move relative to each other, thus displacing the respective secretion signals. However, a covalent attachment of the two domains does not prevent secretion of PelI^70C/240C^, demonstrating that the presumed movements of these two domains, and the related secretion signals, are not essential. This example supports a spatially coordinated pincer-like recognition of the secretion signals located at Fn3 and CD. Interestingly, the superimposition of Pel3 and PelI shows that their respective loops 3 and 5 of Fn3 are apparently displaced in respect to the cognate loop T1.8 of CD (Fig. 3*A*). This indicates that a lack of Pel3 recognition by the *D. dadantii* Out system could be partly due to an improper positioning of these secretion signals relative to each other.

One of the most intriguing observations we made, when comparing the Pel3 and PelI structures, is the presence of a very labile and rare secondary structure in the Fn3 loop3, namely a 3_10_-helix. This helix is located at the exact position that plays a key role in protein recruitment by the T2SS and allows it to discriminate between the Pel3 and PelI. As supporting evidence, when we compared the loop 3 structures in Pel3 and PelI, we observed the presence of an acidic residue in PelI, D58, turned toward the solvent, and replaced by an alanine in Pel3 (A60). This led to the conclusion that the charge difference could be an essential part of the discrimination process. Consistent with this, D58A substitution prevents secretion of PelI by *D. dadantii* (21).

However, a closer inspection of these secondary structures revealed that a more subtle mechanism could be implicated in the recruitment of PelI/Pel3 by cognate T2SS. Indeed, in the different available structures of Pel3, the loop 3 of Fn3 undergoes significant structural variations, from the absence of any stable secondary structure, in the monomer A of Pel3_2m_, to a well-structured 3_10_-helix, in the monomer B of Pel3_2m_ and in Pel3_1m_ (Fig. 4). Stabilization of this labile secondary structure seems to be dependent on interactions with protein partners and neighboring residues. Notably, polar–π interactions between Q59 and W89 residues stabilize the 3_10_-helix in the monomer B of Pel3_2m_ and in Pel3_1m_ (Fig. 4). Therefore, it is tempting to suggest that, in the course of secretion, loop 3 Fn3 could also undergo transient structuration as a result of contact with the appropriate T2SS components.

Interestingly, the Fn3 domain of Pel3/PelI shares more structural similarity with the Fn3 modules from eukaryotic signaling proteins than with those from bacterial carbohydrate-active enzymes. For instance, the Fn3 domains of Pel3 and of human Cell adhesion molecule Downregulated by Oncogenes (CDO) (PDB 3N1F) adopt very similar overall folds with an r.m.s. deviation of 2.0 Å (Fig. S7). Fn3 domains from eukaryotic proteins are involved in various protein–protein interactions by combining the residues from two or three neighboring loops in the binding interface. For example, the loops *CD* and *EF* of human Fn3 CDO (equivalent to loops 3 and 5 of Fn3 Pel3) together form the partner-binding interface with hedgehog signaling protein, Hh (Fig. S12) (34, 35). In addition, depending on the contacts, these loops of Fn3 CDO have been visualized either as unstructured or as carrying a 3_10_-helix supporting the functional relevance of such structuration. Here we show that loops 3 and 5 of Fn3 Pel3 also act together and could constitute a common binding interface with a cognate T2SS component. Taking into account a contact-dependent structuration of the loop 3 Fn3 Pel3 into a 3_10_-helix, these exciting structural similarities suggest some parallels in the interaction mechanisms of these Fn3 domains. Of note is that the eukaryotic Fn3 domains often bind disordered regions of interacting proteins (34, 35, 36). Intriguingly, many T2SS components carry disordered regions in the key positions and their disorder-to-order transitions seem to be functionally relevant (8). Finally, the two other secretion determinants identified in the catalytic domain of Pel3/PelI, namely T3.4 and T1.8, are also variable loop regions, extended over the conserved β-helix domain, and readily accessible for any external contact with cognate T2SS components.

Interestingly, inspection of high-resolution structures of some other T2SS-secreted proteins reveals the presence of some structurally variable zones on otherwise stably folded proteins. For instance, pectate lyase PelC, secreted by the T2SS of *Dickeya*, is composed of a single β-helix domain decorated with several short α-helices and loops. PelC belongs to the PL-1 family and is sequence dissimilar and unrelated to Pel3/PelI (www.cazy.org (26)). Comparison of several crystal structures, available for PelC, reveals some important structural transitions of several exposed protein zones namely loop-to-helix and loop-to-strand (Fig. S13). Such behavior in crystal does not necessarily reflect what could happen *in vivo*, but these structural transitions are in line with our hypothesis.

## Conclusion

The exact nature of the T2S signal is still unclear. Consistent with the highly structured nature of T2S substrates, acquired prior to their recruitment, and the absence of conserved amino acid sequences, a widely shared hypothesis is that such signals could be of a structural nature. However, even the proteins secreted by the same T2SS adopt very different folds, thus complicating the identification of such putative common structural determinants. This study shows that there is not an element with a definite secondary structure but instead there are some unstructured, or intrinsically disordered, segments of the otherwise well-structured protein that could act as a composite secretion signal.

Indeed, it is tempting to speculate that, as observed with the loop 3 Fn3 in the different crystal forms, a contact-induced transient structuration of such intrinsically disordered loop regions could constitute a general mechanism of protein recruitment by the T2SS. This interesting hypothesis is worthy of further exploration with other secreted proteins.

## Experimental procedures

### Strains, plasmids, construction of mutant proteins, and secretion assays

*E. coli* NM522 and BL21(DE3) strains (Stratagene) were used for DNA cloning and production of recombinant proteins, respectively. *D. dadantii* A3756 *pelI:: uidA-nptI* (Km^R^) strain (21) was used for secretion assays. The plasmids and primers used are listed in Tables S4 and S5, respectively. Single and multiple amino acid substitutions were generated by site-directed mutagenesis using PrimeSTAR Max DNA Polymerase (Takara Bio). Sequence of mutant genes was checked in both directions (Eurofins Genomics). To verify if the generated Pel3-PelI hybrid proteins are properly folded and therefore, stable enough in the periplasm, their stability (*i.e.*, persistence in the cells during a prolonged culture incubation) has been tested in the *out* minus strain of *D. dadantii* by immunoblotting with anti-PelI antibodies that cross-react with Pel3 and Pel3-PelI variants (21). Only the hybrids, detected at a level higher than that of Pel3, were retained for secretion assays. Secretion of generated Pel3 variants was estimated by immunoblotting as reported (21). In this order, *D. dadantii pelI* mutant cells carrying pBS plasmid with a Pel3 variant of interest were grown in Luria broth supplemented with 100 μg/ml ampicillin, 0.2% sodium galacturonate, and 0.1% glycerol at 28 °C for 14 to 16 h, till early stationary phase. Culture supernatant containing secreted proteins was separated from cells by centrifugation at 10,000*g* for 3 min, and both fractions were loaded onto 12% polyacrylamide gel electrophoresis (SDS-PAGE) in an equivalent of 0.04 OD_600_ cells. The proteins were next transferred onto Immobilon P membrane (Merck) and probed with rabbit antisera directed against the pectate lyases PelI and PelD (20) and secondary goat anti-rabbit antibodies conjugated to peroxidase (Sigma-Aldrich). PelD was used as a positive secretion control to check if secretion of other T2SS substrates is not affected by expression of Pel3/PelI hybrids. All the secretion assays were repeated at least three times, with two biological replicates (independent cultures). Blots were developed with Luminata forte substrate (Millipore) and chemiluminescence signal was recorded and quantified with Vilber fusion FX6 imaging system (Vilber Lourmat) or exposed to Hyperfilm (GE Healthcare). For each Pel3/PelI hybrid, the percentage of secreted protein relative to the sum of the corresponding hybrid protein in culture supernatant and cells was estimated as S/(S + C). Six to 10 scan quantifications from three independent experiments were performed with each hybrid, and the mean values ± SD were shown on Figure 3*C*.

### Protein purification

The sequence of the mature Pel3 (residues Asp22 to Leu347) was fused to that of the PelB signal peptide onto pET-20b(+) vector (Novagen) and expressed in the periplasm of *E. coli* BL21 (DE3) cells (Stratagene). The bacteria were grown in Luria broth supplemented with 150 μg/ml ampicillin at 30 °C to an OD_600_ of 0.6 next, induced with 1 mM IPTG (isopropyl-β-d-thiogalactopyranoside), and grown for an additional 3 h. The cells were frozen at −80 °C and the mature Pel3 protein (signal peptide-less) was extracted by three cycles of freezing–thawing as described previously (45, 46). Pel3 was next precipitated with ammonium sulfate (60–90% step saturation) for 24 h at 4 °C. After centrifugation at 5000*g* for 30 min, the protein pellet was solubilized in 20 mM sodium phosphate buffer pH 7, 0.1 mM EDTA, 1.7 M ammonium sulfate (Buffer A) and loaded onto TSKgel-Phenyl column (Tosoh Bioscience) equilibrated with the same buffer. The column was next washed with Buffer A and protein was eluted by applying a gradient of ammonium sulfate from 1.7 to 0.8 M in 20 mM sodium phosphate buffer pH 7, 0.1 mM EDTA. Fractions containing Pel3 were collected and subjected to ion-exchange chromatography using Hi-Trap Sulfopropyl-Sepharose column (GE Healthcare) equilibrated in 20 mM sodium acetate buffer pH 5, 0.1 mM EDTA (Buffer B). The column was washed with buffer B and Pel3 protein was eluted by charge inversion with 20 mM Tris buffer pH 8.8, 0.1 mM EDTA by applying a NaCl gradient from 0 to 100 mM. Pure protein fractions were concentrated with Vivaspin devices (Sartorius) to 20 mg/ml and used in crystallization trials.

### Crystallization

Crystallization screening was carried out at 293K (vapor-diffusion by the sitting-drop method), with commercially available crystallization kits. For screening, a mosquito crystallization robot from TTP Labtech was employed (150 nl + 150 nl drops equilibrated against 70 μl). Pel3 crystals suitable for X-ray analysis grew within 3 months either in a solution containing 1 M succinic acid, 0.1 M HEPES pH 7.0, 1% (m/v) PEG MME 2000 (4U4B) or 0.2 M lithium acetate, 20% (w/v) PEG3350 (4U49). For cryoprotection, crystals were soaked in a reservoir solution supplemented with 15% (v/v) ethylene glycol during 2 to 5 min. X-ray diffraction data were collected at beamline X06DA at the SLS (Paul Scherrer Institute, Switzerland), at a wavelength of 0.979 Å for 4U49 (Pel3_2m_), or at beamline ID29 at the ESRF (Grenoble, France), at a wavelength of 0.939 Å for 4U4B (Pel3_1m_). Pel3 crystals diffracted X-rays to 2.10 Å resolution for 4U4B and 1.80 Å resolution for 4U49. They were both indexed and scaled with the program XDS. The crystal structure of Pel3 was solved by the molecular replacement method with the program PHENIX-AutoBuild (47) using the refined structure of PelI from *D. dadantii* (PDB 3B4N) as search model. The structure was then refined with the program PHENIX-Phaser and visualized with COOT software (48). The refined structure was validated with PROCHECK (49) before depositing.

### Phylogenetic analysis

Candidate homologs were searched at a protein level by using BLAST (50) and Pel3 as a query sequence. Search was done against the UniProtKB database. Three different BLAST searches were done, respectively, against Bacteria, Fungi, and Nematodes. All sequences that aligned at least on 80% of the length of the Pel3 catalytic domain were kept for multiple sequence alignment. Sequences were then clustered with CD-HIT in order to keep a significant number of sequences for each group. Some other sequences were then manually added, namely two oomycota sequences from NCBI database (entries XP_024585360_1 and XP_024581403_1), since only two oomycota representatives are available in UniprotKB database (September 2020) and three sequences corresponding to the available protein structures from the PL-3 family (entries O50325, B9MKT4, and Q9RHW0, corresponding to the PDB entries 3B4N, 3T9G, and 1EE6, respectively). Multiple sequence alignments were done with MAFFT program (51) and conserved blocks were selected by using BMGE 1.12 (52), using the BLOSUM30 (53) matrix with both programs. In total, 154 sites were kept for further analysis after the character trimming method performed by BMGE (52). Phylogenetic analyses were performed, with the LG model and a gamma correction, using two approaches: a Bayesian method with mrBayes (54) and a bootstrapped maximum-likelihood approach with PhyML (55). By default, 100,000 generations were run when using mrBayes, and we ran 100 bootstrap replicates when using PhyML. Phylogenetic tree was generated and visualized with iTOL software (56).

## Data availability

All data described in the article are contained within the article. Pel3 structure coordinates have been deposited at the Protein Data Bank in Europe (PDBe) database with accession codes 4U49 and 4U4B. Strains and plasmids described in this article are available upon request from Vladimir Shevchik (vladimir.shevchik@insa-lyon.fr).

## Conflict of interest

The authors declare that they have no conflicts of interest with the contents of this article.
