# Supplementary material for: Structure–function analysis of pectate lyase Pel3 reveals essential facets of protein recognition by the bacterial type 2 secretion system
Source: J Biol Chem. 2021 Jan 16;296:100305. doi: 10.1016/j.jbc.2021.100305 (PMC7949064; doi:10.1016/j.jbc.2021.100305)
Supplement: Supplemental Figures and Tables [file mmc1.pdf]

Supporting information

Structure-function analysis of pectate lyase Pel3 reveals essential facets of protein recognition by the bacterial type 2 secretion system.

Camille Pineau, Natalia Guschinskaya, Isabelle Gonçalves, Florence Ruaudel, Xavier Robert, Patrice Gouet, Lionel Ballut, Vladimir E. Shevchik

Supporting figures

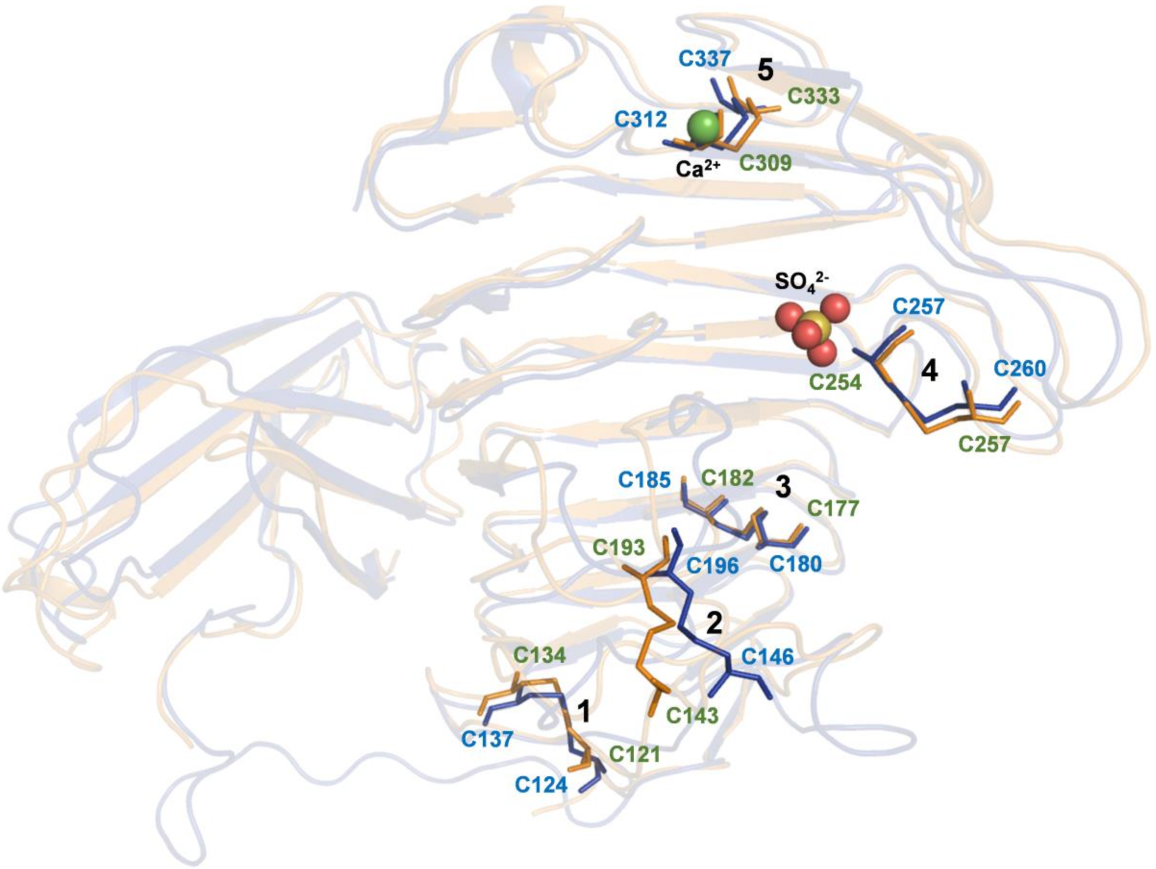

Figure S1. Conservation of the disulfide bonds in Pel3 and PelI. Superposition of Pel3 (blue, PDB 4U49) with PelI (orange, PDB 3B4N) shown in transparency. The conserved cysteine residues forming disulfide bonds in the catalytic domain are shown as bold sticks. The disulfide bonds are numbered from 1 to 5 as in the main text. Bound calcium and sulfate ions are shown for Pel3.

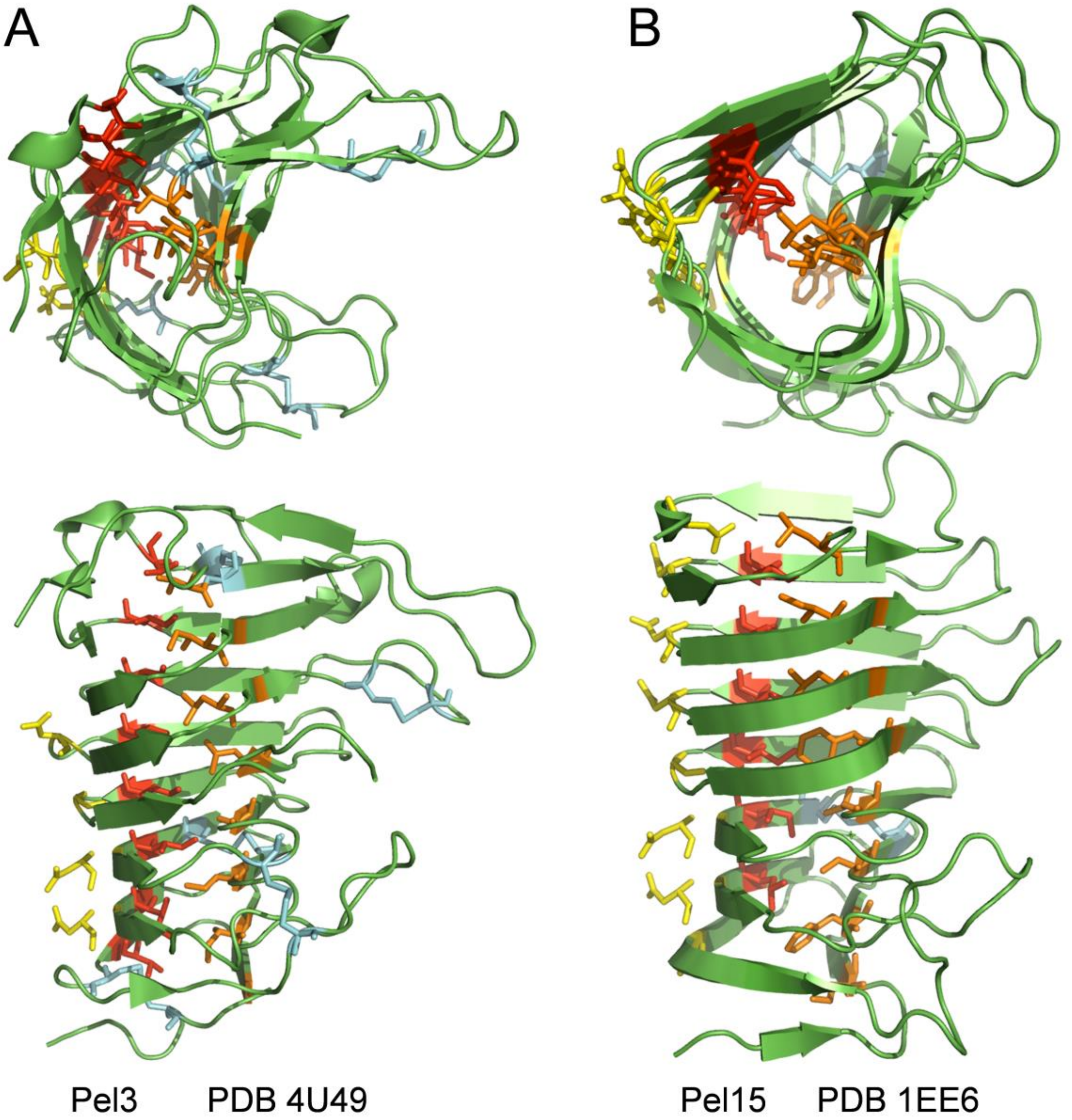

Figure S2. The PL-3 β-helix is stabilized by a series of hydrophobic stacking and an Asn ladder. A and **B**, bottom and lateral views of Pel3 from *P. carotovorum* (PDB 4U49) and Pel-15 from *Bacillus sp.* KSM-P15 (PDB 1EE6). The hydrophobic core of β-helices is stabilized by a series of hydrophobic interactions between the inward-pointing side chains of several aliphatic residues and Phe. These residues are organized into two regular ladders extending along the β-strands PB1 and PB3, in orange and red, respectively. The Pel-15 structure (B) is additionally stabilized by an Asn ladder (in yellow), extending along the whole length of the β-helix. Only a few Asn residues of this ladder are conserved in Pel3 (A). Note that the Pel3 β-helix is apparently less regular than that of Pel-15 and carries some additional loops, short helices and strands. Five disulfide bonds (in cyan) further reinforce the basic β-helix framework and extended loops of Pel3.

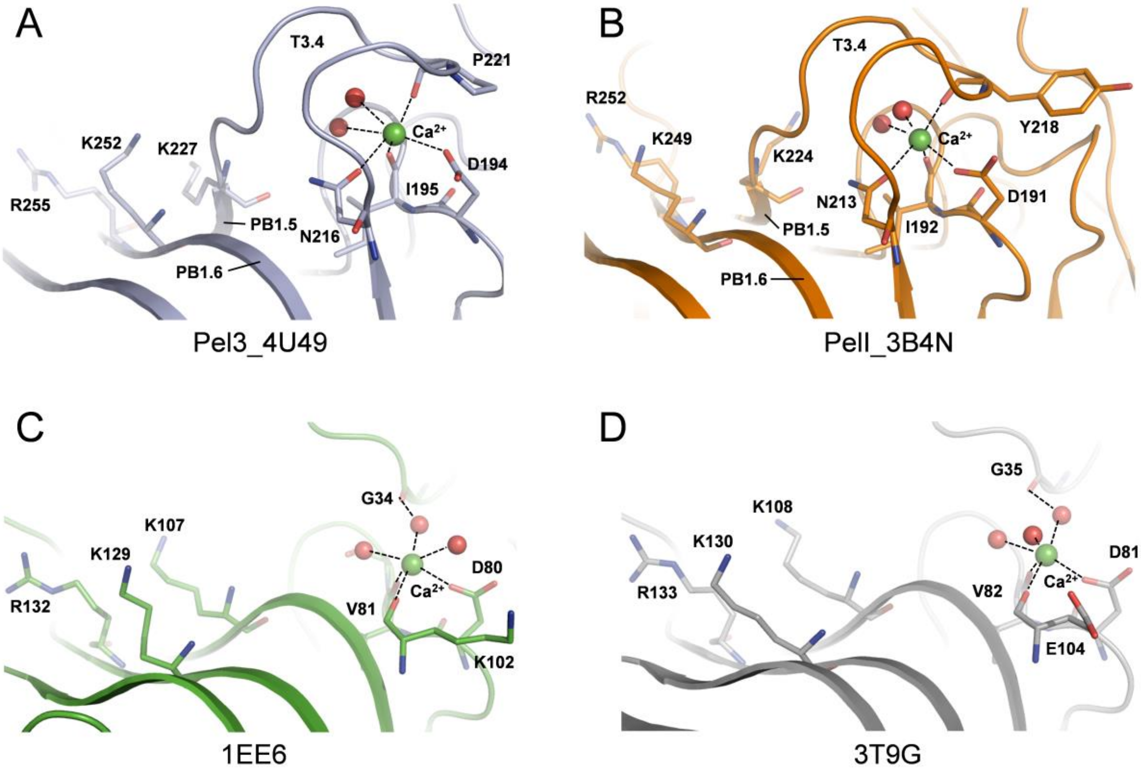

Figure S3. Catalytic site conservation in the PL-3 family proteins. A and **B,** The invariant residues of Pel3 and PelI present in or adjacent to the catalytic site. Lys^{227} (Pel3) and Lys^{224} (PelI) represent the catalytic base. Lys^{252} and Arg^{255} (Pel3) or Lys^{249} and Arg^{252} (PelI) are implicated in the binding of the substrate. Side chain of Asp^{194} and Asn^{216} (Pel3) or Asp^{191} and Asn^{213} (PelI) as well as main chain of Ile^{195} and Pro^{221} (Pel3) or Ile^{192} and Tyr^{218} (PelI) plus two water molecules are implicated in calcium coordination and stabilization of loop T3.4. **C** and **D,** In PL-3 from *Bacillus sp.* KSM-P15 (PDB 1EE6) and *C. bescii* (PDB 3T9G), the catalytic residues equivalent to Lys^{227}, Lys^{252} and Arg^{255} of Pel3 as well as Ca-binding Asp are conserved but loop equivalent to T3.4 is absent. In 1EE6 and 3T9G, the main chain carbonyl of Pro^{221} (Pel3) and Tyr^{218} (PelI) carried by loop T3.4 are replaced by a water molecule.

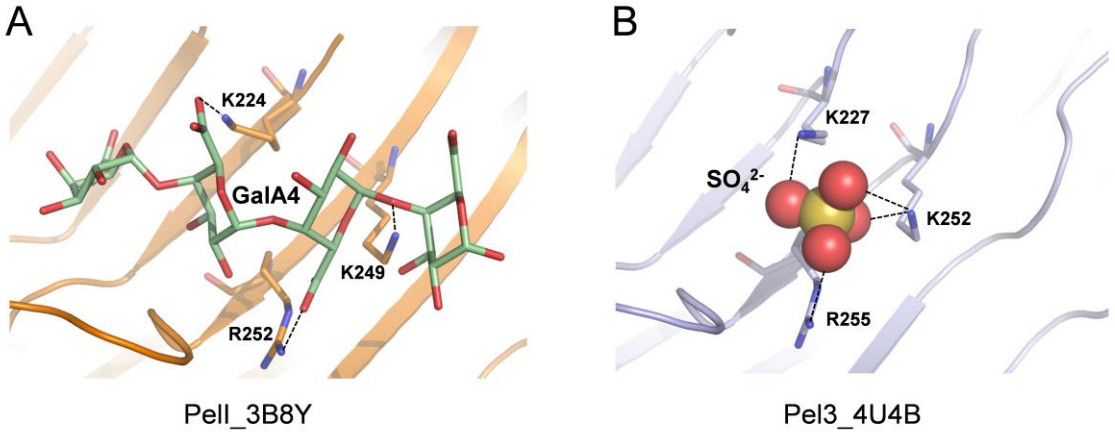

Figure S4. Sulfate ion mimics the substrate in the catalytic site of Pel3. Sulfate ion in Pel3 (B) forms salt bridges with Lys^{227}, Lys^{252} and Arg^{255}, mimicking hydroxyl groups of tetragalacturonic acid present in the catalytic site of PelI (A).

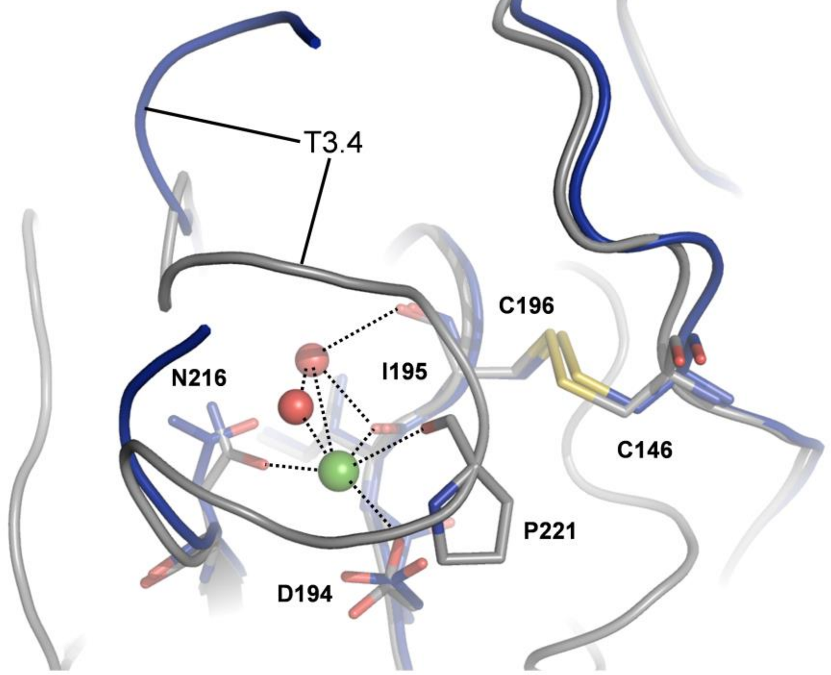

Figure S5. Destabilization of loop T3.4 in the catalytic domain of Pel3 in the absence of calcium. Superposition of the loop T3.4 and neighbor regions from monomer A (blue) and monomer B (grey) of $Pel3_{2m}$ PDB 4U49. The residues coordinating Ca ion in PDB_{1m} and disulfide bond Cys_{146}-Cys_{196} are shown. In monomer A, the loop T3.4 is destabilized and not visible.

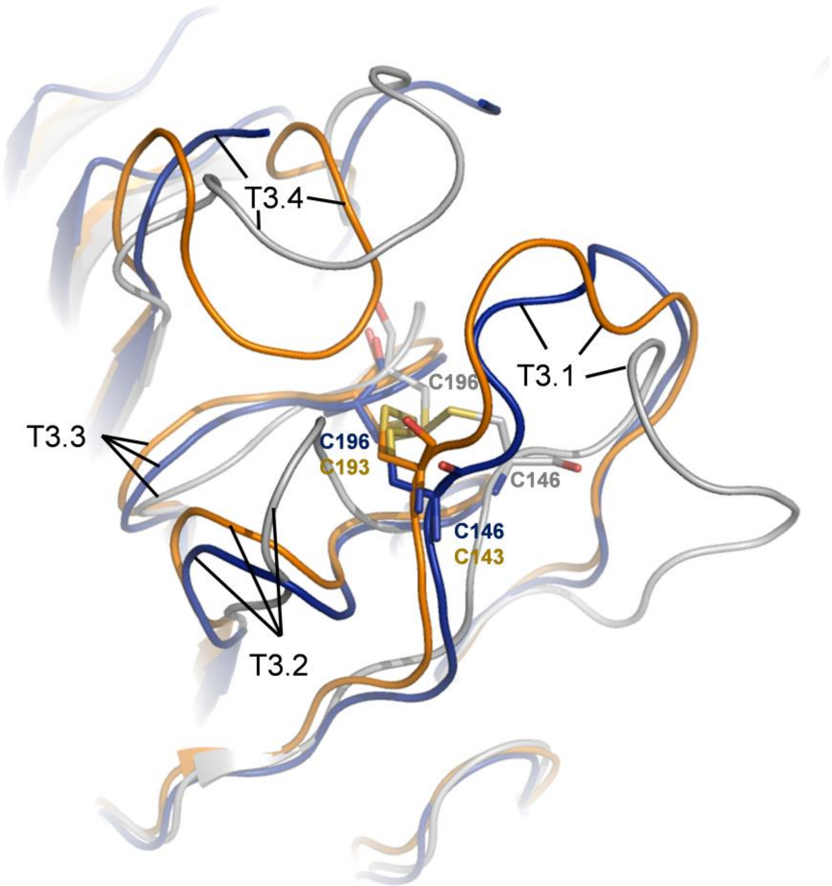

Figure S6. Loops T3.1, T3.2 and T3.4 are differently arranged in various structures of Pel3 and PelI. Superposition of the catalytic domains from $Pel3_{1m}$ (grey, PDB code 4U4B), $Pel3_{2m}$ monomer A (blue, PDB code 4U49) and PelI (orange, PDB code 3B4N): only the protein zones with the exposed loops of interest are shown. Of note, the disulfide bond attaching the loops T3.1 and T3.3 is also displaced between $Pel3_{1m}$ and $Pel3_{2m}$ structures.

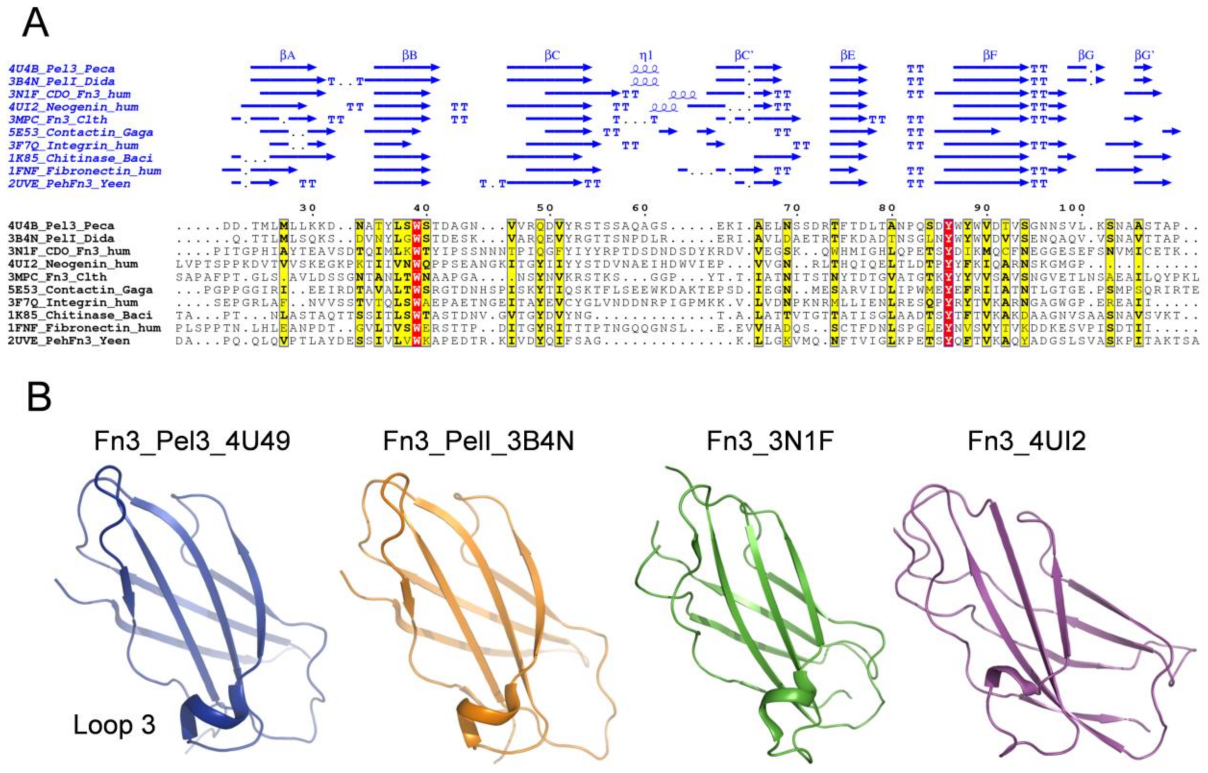

Figure S7. Fn3 domain of Pel3 is structurally similar to Fn3 domains from eukaryotic signaling proteins. A, Structure-based sequence alignment of Fn3 domain from Pel3 with its structural homologs identified using the Dali server (Table S3). The PDB codes are 4U4B, pectate lyase Pel3 from *Pectobacterium carotovorum*; 3B4N, pectate lyase PelI from *Dickeya dadantii*; 3N1F, Fn3 domain from human Cell adhesion molecule Down-regulated by Oncogenes (CDO); 4UI2, 5th Fn3 domain from human neogenin; 3MPC, Fn3-like protein from *Clostridium thermocellum*; 5E53, Fn3 domain from chicken Contactin-1; 3F7Q, Fn3 domain from human integrin β4; 1K85, Fn3 domain from *Bacillus circulans* chitinase; 1FNF, Fn3 domain from human fibronectin; 2UVE, Fn3 domain from *Yersinia enterocolitica* exopolygalacturonase. The secondary structure elements are shown for each protein. The conserved protein zones are in yellow and red boxes, for similar and identical residues, respectively. **B,** Overall structure conservation between Fn3 domains of Pel3 (PDB 4U49, blue), PelI (PDB 3B4N, orange), CDO (PDB 3N1F, green) and cell surface receptor neogenin NEO1 (PDB 4UI2, violet). Of note, a helix is present in the same place in loop 3 of Pel3 and PelI as well as in two eukaryotic Fn3 domains.

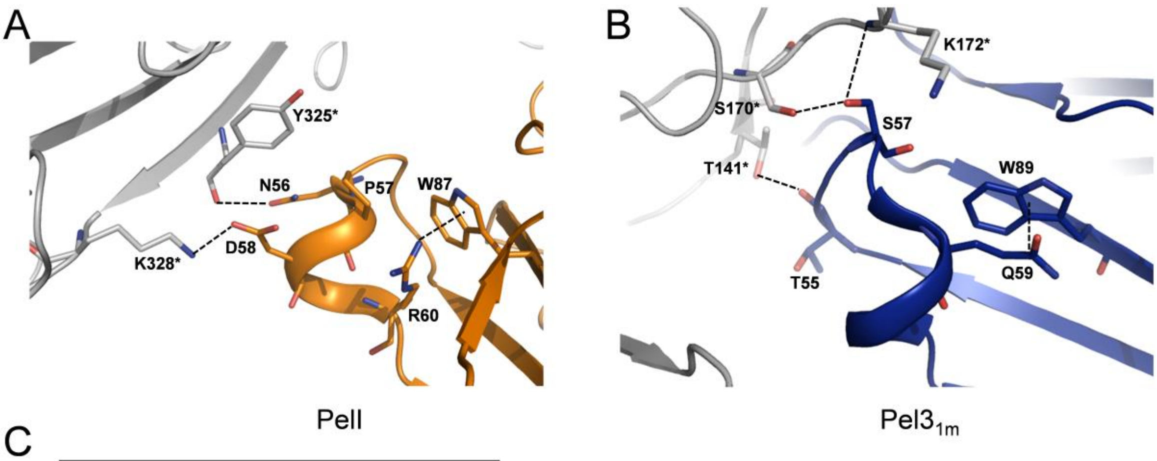

C

3_{10} helix			
Angles	P57	D58	L59
Phi	-60.94	-66.44	-101.86
Psi	-27.96	-17.75	0.39

Figure S8. Stabilization of loop 3 Fn3 in PelI and Pel3. A and **B**, Close up view of the protein zones around of loop 3 Fn3 of PelI (orange, monomer A in PDB 3B4N) and $Pel3_{1m}$ (blue, PDB 4U4B). The orientation shows all the interactions between PelI and Pel3 and their respective crystal neighbor in grey (residues numbering with asterisks). The polar π interaction between Gln59 and Trp89 in Pel3 is replaced by a cation π interaction between Arg60 and Trp87 in PelI. **C**, phi and psi angles of residues Pro57, Asp58 and Leu59 forming 3_{10} helix in loop 3 of Fn3 PelI.

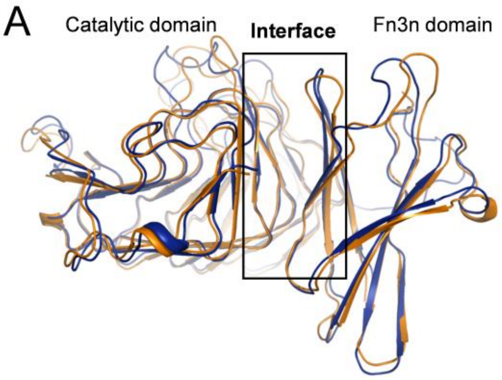

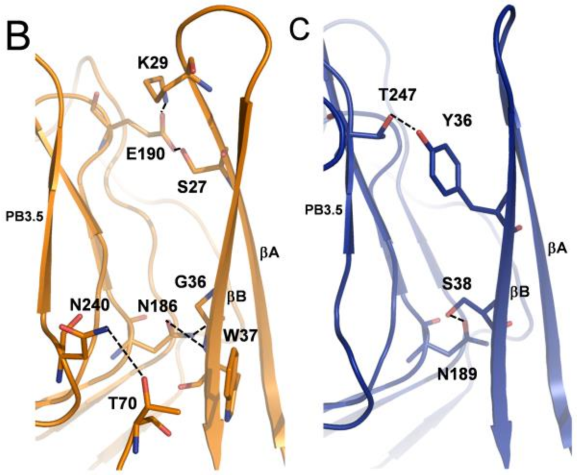

D

Pel I			
H bonds	Fn3 domain	Distance [Å]	Catalytic domain
1	Thr 23 [O]	3.79	Asn 186 [ND2]
2	Leu 26 [O]	2.96	Arg 165 [NH1]
3	Leu 26 [O]	2.86	Arg 165 [NH2]
4	Tyr 34 [OH]	2.51	Asn 274 [ND2]
5	Ser 38 [OH]	2.76	Asn 163 [OD1]
6	Arg 71 [OH]	2.87	Gly 239 [O]
7	Thr 72 [N]	3.90	Gly 239 [O]
8	Thr 72 [OG1]	2.80	Thr 242 [OG1]
9	Thr 72 [OG1]	3.34	Asn 240 [O]
10	Thr 72 [OG1]	3.23	Asn 240 [ND2]
**11**	**Ser 27 [OG]**	**2.97**	**Glu 190 [OE2]**
**12**	**Gly 36 [O]**	**3.14**	**Asn 186 [ND2]**
**13**	**Trp 37 [N]**	**3.20**	**Asn 186 [OD1]**
**14**	**Thr 70 [OG1]**	**3.44**	**Asn 240 [ND2]**
Salt bridges			
**1**	**Lys 29 [NZ]**	**2.63**	**Glu 190 [OE1]**

E

Pel 3			
H bonds	Fn3 domain	Distance [Å]	Catalytic domain
1	Met 25 [O]	3.18	Asn 189 [ND2]
2	Leu 28 [O]	3.15	Arg 168 [NH1]
3	Leu 28 [O]	2.84	Arg 168 [NH2]
4	Tyr 36 [OH]	2.80	Asn 277 [ND2]
5	Ser 40 [OH]	2.78	Asn 166 [OD1]
6	Arg 73 [OH]	3.00	Gly 242 [O]
7	Thr 74 [N]	3.53	Gly 242 [O]
8	Thr 74 [OG1]	3.37	Thr 245 [OG1]
9	Thr 74 [OG1]	2.79	Asn 243 [O]
10	Thr 74 [OG1]	3.46	Asn 243 [ND2]
**11**	**Tyr 36 [OH]**	**2.56**	**Thr 247 [OG1]**
**12**	**Ser 38 [OG]**	**2.51**	**Asn 189 [OD1]**

Figure S9. Organization of the interface between Fn3 and catalytic domains of Pel3 and PelI. A, superposition of Pel3 (blue, PDB 4U49) with PelI (orange, PDB 3B4N) with the catalytic and Fn3 domains arranged around the interface. **B** and **C,** close up view of interdomain interfaces of PelI and Pel3 showing polar and ionic interactions specific for each protein. **D** and **E**, The distance of H bonds and salts bridges observed respectively, in the interface of PelI and Pel3. Interactions specific for each protein are in bold.

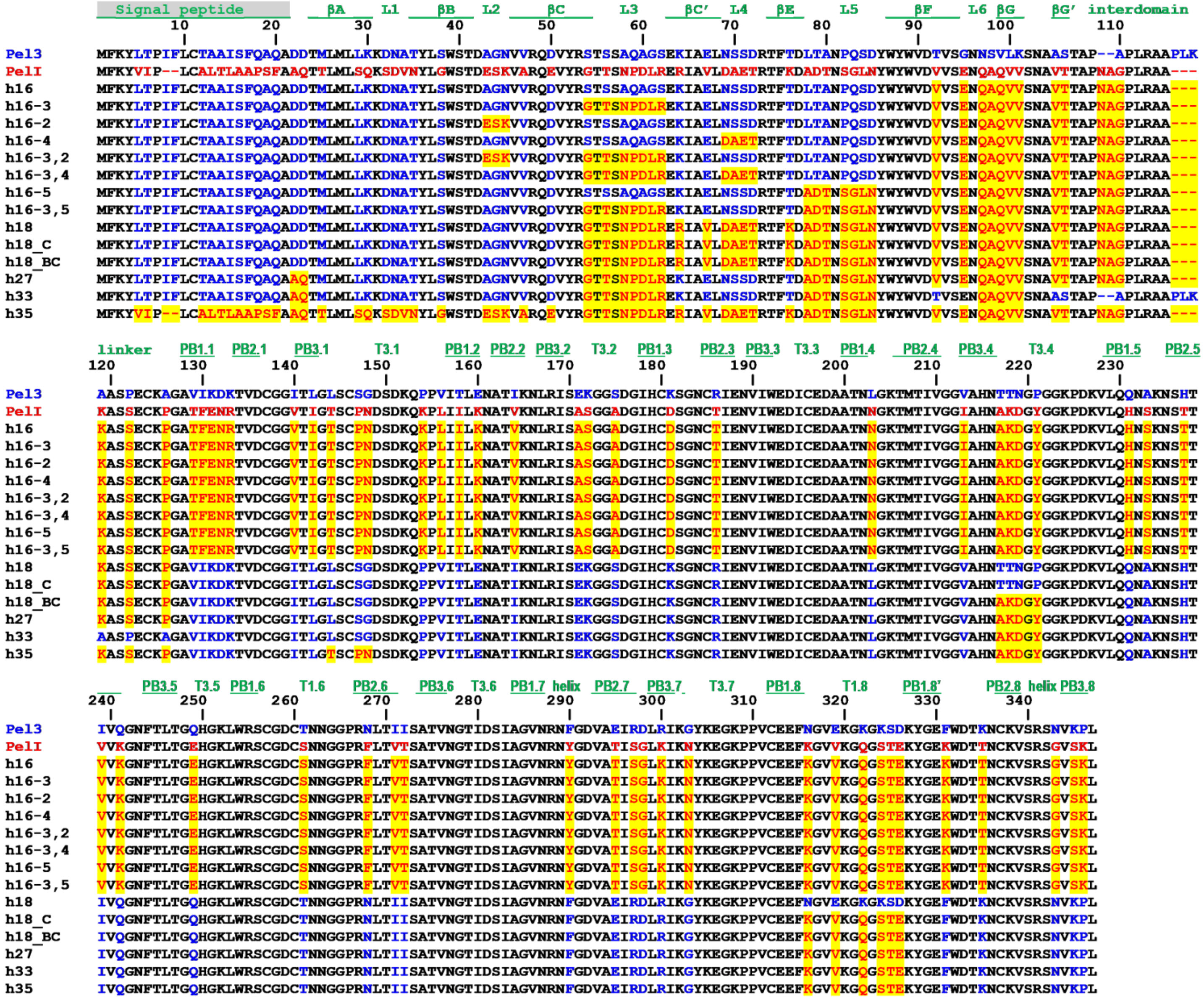

Figure S10. Sequence alignment of Pel3-PelI variants used in this study. The secondary structure elements are shown for *P. carotovorum* Pel3 (PDB 4U4B). The residue numbering is shown for the immature Pel3 polypeptide carrying a signal peptide. The residues conserved between Pel3 and PelI are in black while these specific for Pel3 and PelI are in blue and in red, respectively. The residues substituted in hybrid proteins are highlighted in yellow.

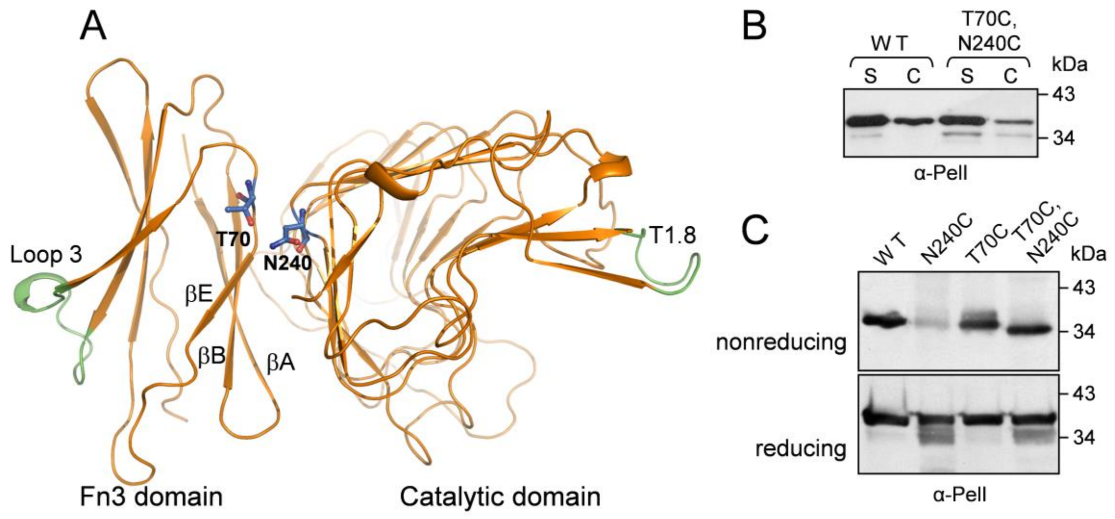

Figure S11. Locking of the interdomain Fn3/CD interface by disulfide bond does not prevent secretion of PelI. **A**, Close up view of the PelI interface: the residues substituted with cysteine generating an interdomain cross-linking are shown as blue sticks and loops 3 and T1.8 are in green. **B**, Secretion efficiency of the wild-type PelI and T70C/N240C variant expressed from a plasmid in *D. dadantii* A5159 *pelI* strain: immunodetection in cell extract (C) and culture supernatant (S) with antibodies raised against PelI. **C**, Electrophoretic mobility assay. The wild type PelI, the single and double cysteine variants were separated on reducing and non-reducing gels (with and without 2-mercapthoethanol, respectively) and visualized by immunoblotting with anti-PelI. Note that in non-reducing conditions, $PelI^{T70C/N240C}$ runs slightly faster than $PelI^{WT}$ indicating a more compact overall shape imposed by the interdomain disulfide bond.

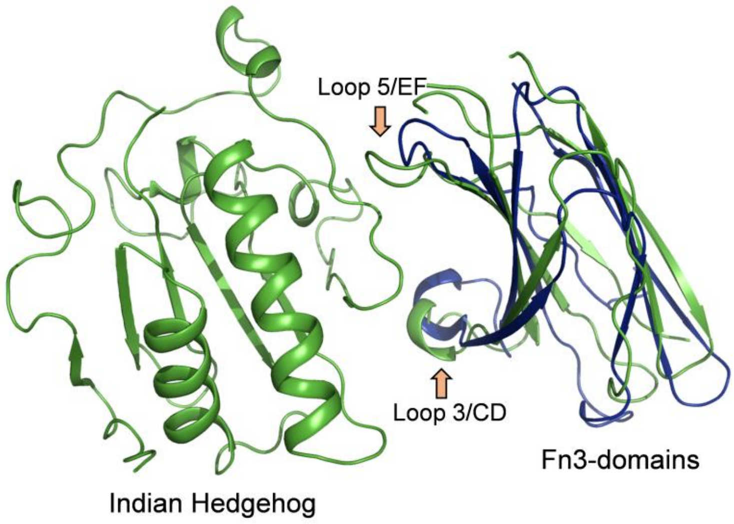

Figure S12. Superposition of the Fn3 domains from human Cell adhesion molecule, Down-regulated by Oncogenes (CDO) (PDB 3N1F, green) and Pel3$_{2m}$ monomer B (PDB code 4U4B, blue). Human CDO Fn3 interacts with Indian Hedgehog protein (in left): the binding interface involves loops CD and EF of CDO Fn3 (equivalent to loops 3 and 5 of Fn3 Pel3) and the linker from Indian Hedgehog protein.

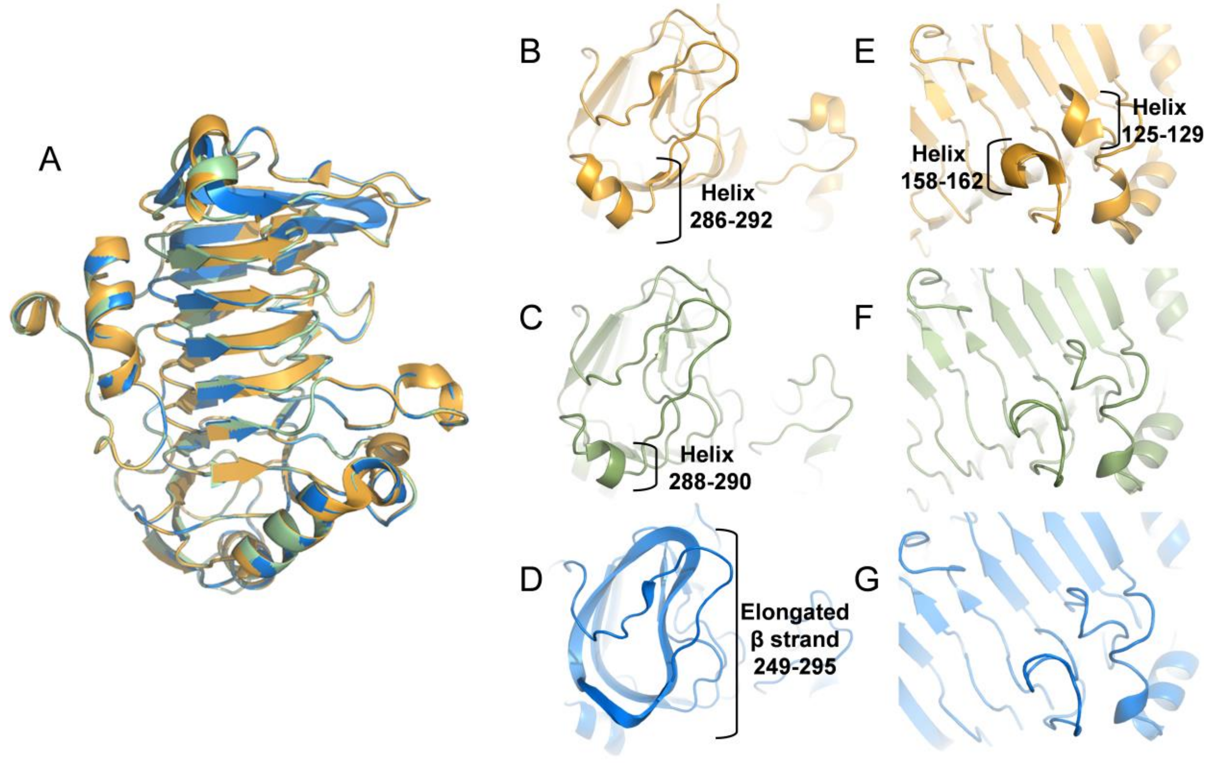

Fig S13. Structural variations of loop regions in different structures of pectate lyase PelC. A, Overall structure conservation between three different structures of PelC from *Dickeya chrysanthemi* (formerly *Erwinia chrysanthemi*) (PDB entries 1O8I (orange), 2PEC (green) and 1AIR (blue) are superimposed). **B** to **G**, Close up view showing loop-to-helix or loop-to-β strand transition or helix reorganization of indicated protein zones. **B** to **D**, Helix (residues 286-292) in 1O8I (B) is shortened in 2PEC (C) and a large protein region including this zone is restructured into a long beta strand in 1AIR (D). **E** to **G**, Two helices (residues 125-129 and 158-162) in 1O8I (E) are restructured in loops in 2PEC (F) and 1AIR (G).

Supporting Tables

Table S1. Data collection and refinement statistics

Data collection		
**Code PDB**	**4U4B**	**4U49**
Synchrotron beamline	ID29, ESRF	X06DA, SLS
Wavelength (Å)	0.94	0.98
Space group	$P2_1$	$P2_1$
Unit-cell parameters (Å,°)	*a* = 37.0, *b* = 44.3, *c* = 86.2, β = 93.6	*a* = 48.6, *b* = 72.0, *c* = 83.7, β = 101.3
Resolution limit (Å)	2.1 (2.2-2.1)	1.8 (1.9-1.8)
Number of measurements	43,537(3,021)	177,066(25,948)
Unique reflections	15,116 (1,389)	52,416 (7,784)
Completeness (%)	91.6(65.0)	99.8(99.8)
R_{meas} (I) [a](%)	5.4 (12.7)	5.1 (49.1)
CC(1/2)[b](%)	99.7(97.3)	92.4(82.0)
Mean I/σ(I)	19.2 (7.3)	16.9 (2.8)
**Crystallographic refinement**		
Asymmetric unit content	1 monomer	2 monomers
Number of non- hydrogen protein atoms	2,630	5,479
Number of water molecules	183	772
Other heteroatoms	9	1
Mean *B*-factor (Å²)	21.0	25.0
R_{factor}/ R_{free}[c] (%)	17.0/25.0	17.7/21.4
Stereochemical quality of the model		
RMSD bond lengths (Å)	0.008	0.003
RMSD bond angle (°)	1.1	0.8
Ramachandran plot favored (%)	92.3	94.2
Ramachandran outliers (%)	0.3	0

Values in parentheses are for the high-resolution shell. ESRF, European Synchrotron Radiation Facility; SLS, Swiss Light Source; RMSD, root mean square deviation.

[a] $R_{meas} = \sum_{hkl}[N/(N-1)]^{1/2} \sum_i |I_i(hkl) - \langle I(hkl)\rangle| / \sum_{hkl} \sum_i I_i(hkl)$, where *N* is the multiplicity of a given reflection.

[b] CC(1/2) values were calculated using the program XDS.

[c] R_{factor} and R_{free} are given by $\sum|F_{obs} - F_{calc}|/\sum F_{obs}$ with $R_{free} = R_{work}$ calculated using 5% random data excluded from the refinement.

Table S2. Protein sequences used to generate the phylogenetic tree of Pel3 homologs.

Accession number	Group	Name	Species	Mutations, secretion systems	Disulfides and cysteine*	N terminal domain
Proteobacteria & Firmicutes (enzymes)						
B9MKT4_3T9G	Firmicutes	Cabe	*Caldicellulosiruptor bescii*		5 Cys, No disulfides	CBM
A0A285I5J8	Firmicutes	Orme	*Orenia metallireducens*		3 Cys	Ricin B like
Q9RHW0_1EE6	Firmicutes	Basp	*Bacillus sp* KSM-P15		#3 + 1 Cys	No
Q9X6Z2	Firmicutes	Paba	*Paenibacillus barcinonensis*		#3*	No
A0A2E3CYN3	γ Proteobacteria	Psba	*Pseudomonadales bacterium*		6 Cys, 1 unusual disulfide*	Ricin B like
A0A2D8RH27	γ Proteobacteria	Haba	*Hahellaceae bacterium*		6 Cys, 1 unusual disulfide*	Ricin B like
A0A085C4I9	Firmicutes	Basu	*Bacillus subtilis*		1 Cys	No
A0A1H2VLD4	Firmicutes	Pasp	*Paenibacillus sp. PDC88*		No Cys	Ricin B like
A0A2L0F5X3	δ Proteobacteria	Soce	*Sorangium cellulosum*		No Cys	CBM lipoprotein
Proteobacteria (HrpW)						
A0A246SKK6	α Proteobacteria	Rhsp	*Rhizobium sp. R635*	T3SS, K252T	No Cys	Nt disorder zone
A0A1I7LW60	β Proteobacteria	Mana	*Massilia namucuonensis*	T3SS	No Cys	G,S rich disorder
A0A1I1V4D4	β Proteobacteria	Acko	*Acidovorax konjaci*	T3SS	No Cys	G,S rich disorder
A0A1I6LAK2	β Proteobacteria	Misp	*Mitsuaria sp. PDC51*		No Cys	G,S rich disorder
F2JTB7	γ Proteobacteria	Mame	*Marinomonas mediterranea*	T3SS	No Cys	G,S rich disorder
A8E411	γ Proteobacteria	Psvi	*Pseudomonas viridiflava*	T3SS, K252T	No Cys	Gly rich disorder
J3GJW8	γ Proteobacteria	Pssp	*Pseudomonas sp. GM50*	T3SS, K252T	No Cys	G,S rich disorder
J3DN95	γ Proteobacteria	Pssp	*Pseudomonas sp. GM102*	T3SS, K252T	No Cys	G,S rich disorder
E0SCP0	γ Proteobacteria	Dida	*Dickeya dadantii 3937*	T3SS, K252T	No Cys	G,S rich disorder
A0A2K8QM43	γ Proteobacteria	Difa	*Dickeya fangzhongdai*	T3SS, K252T	No Cys	G,S rich disorder
Q6RK52	γ Proteobacteria	Peat	*Pectobacterium atrosepticum*	T3SS, K252T	No Cys	G,S rich disorder
A0A221TA52	γ Proteobacteria	Peca	*Pectobacterium carotovorum*	T3SS, K252T	No Cys	G,S rich disorder
Actinobacteria II						
A0A2S5VX08	Actinobacteria	Clmi	*Clavibacter michiganensis*	K252T	#3, 4 and 5	N-terminal TMS

A5CLX7	Actinobacteria	Clmi	*Clavibacter michiganensis*	K252S	#3, 4 and 5	N-terminal extension
B0RG35	Actinobacteria	Clmi	*Clavibacter sepedonicus*	K252T	#3, 4 and 5	No
Nematodes II						
A0A023NDI5	Nematoda	Glro	*Globodera rostochiensis*		#3, 4, 5 + 3 Cys	No
Q53EK1	Nematoda	Glro	*Globodera rostochiensis*		#3, 4, 5 + 3 Cys	No
H6SWR2	Nematoda	Hegl	*Heterodera glycines*		#3, 4, 5 + 3 Cys	No
H6SWR1	Nematoda	Hegl	*Heterodera glycines*		#3, 4, 5 + 3 Cys	No
H6SWR0	Nematoda	Hegl	*Heterodera glycines*		#3, 4, 5 + 3 Cys	No
A3F5B9	Nematoda	Hesc	*Heterodera schachtii*		#3, 4, 5 + 3 Cys	No
H6SWR3	Nematoda	Hegl	*Heterodera glycines*		#3, 4, 5 + 3 Cys	No
Nematodes III						
E5D240	Nematoda	Heav	*Heterodera avenae*	R255C	#2, 3, 4, 5 + 3 Cys	FD-like domain
F2YA46	Nematoda	Glpa	*Globodera pallida*	R255C	#2, 3, 4, 5 + 3 Cys	No
A3F5C0	Nematoda	Hesc	*Heterodera schachtii*	R255C	#2, 3, 4, 5 + 3 Cys	No
Proteobacteria (Pel3)						
S9PKB9	δ Proteobacteria	Cyfu	*Cystobacter fuscus DSM2262*	T2SS	#2, 3, 4 and 5	No, lipoprotein signal
A0A2N4XV85	β Proteobacteria	Ulsp	*Uliginosibacterium sp. TH139*	T2SS	#1, 2, 3, 4 and 5	60 residue long, 4 Cys
A0A2N4XNH5	β Proteobacteria	Ulsp	*Uliginosibacterium sp. TH139*	T2SS	#1, 2, 3, 4 and 5	60 residue long, 4 Cys
A0A437JHM8	γ Proteobacteria	Rhpa	*Rheinheimera pacifica*	T2SS	#1, 2, 3, 4 and 5	Fn3
A0A1M5WEI0	γ Proteobacteria	Viae	*Vibrio aerogenes CECT 7868*	T2SS	#1, 2, 3, 4 and 5	Fn3
A0A1Y6BEP8	γ Proteobacteria	Alba	*Alteromonadaceae bacterium Bs31*	T2SS	#1, 2, 3, 4 and 5	Fn3 + two CBM
A0A1G9AB09	γ Proteobacteria	Psin	*Pseudomonas indica*	T2SS	#1, 2, 3, 4 and 5	two Fn3
Q47465	γ Proteobacteria	Peca_Pel3	*Pectobacterium carotovorum*	T2SS	#1, 2, 3, 4 and 5	Fn3
O50325	γ Proteobacteria	Dida_PelI	*Dickeya dadantii*	T2SS	#1, 2, 3, 4 and 5	Fn3
Nematodes I						
M4VRF2	Nematoda	Buxy	*Bursaphelenchus xylophilus*	K252R	#3, 4, 5 + 2 Cys	No
Q33CQ0	Nematoda	Bumu	*Bursaphelenchus mucronatus*		#3, 4 and 5	No
Q33CQ3	Nematoda	Buxy	*Bursaphelenchus xylophilus*		#3, 4 and 5	No
Q33CQ1	Nematoda	Bumu	*Bursaphelenchus mucronatus*		#3, 4 and 5	No
Q33CQ4	Nematoda	Buxy	*Bursaphelenchus xylophilus*		#3, 4 and 5	No
Actinobacteria I						
C6WMH1	Actinobacteria	Acmi	*Actinosynnema mirum*		#3, 4 and 5	No

A0A0X3V446	Actinobacteria	Acaw	*Actinoplanes awajinensis subsp. mycoplanecinus*		#3, 4 and 5	TAT signal + CBM
A0A2R4JZH5	Actinobacteria	Stsp	*Streptomyces sp. P3*	K252E	#3, 4 and 5	TAT signal
A0A0A0B7Q7	Actinobacteria	Cece	*Cellulomonas cellasea*		#3, 4 and 5	RicinB
A0A162JQF4	Actinobacteria	Frsp	*Frankia sp. EI5c*		#3, 4 and 5	CBM22
A0A2R4FVW9	Actinobacteria	Plsp	*Plantactinospora sp. BB1*	K252T	#3, 4, 5 + 2 Cys	Ricin B like
A0A136PXY2	Actinobacteria	Miro	*Micromonospora rosaria*		#3, 4, 5 + 2 Cys	Ricin B like
A0A1Q4X116	Actinobacteria	Sasp	*Saccharothrix sp. CB00851*		#3, 4 and 5	Ricin B like
Nematodes IV						
A0A0H3U5M6	Nematoda	Megr	*Meloidogyne graminicola*	K252R	#3, 4, 5 + 3 Cys (1 unusual disulfide*)	No
E2JE18	Nematoda	Meen	*Meloidogyne enterolobii*	K252R	#3, 4, 5 + 6 Cys (1 unusual disulfide*)	No
Q7YW99	Nematoda	Mein	*Meloidogyne incognita*	K252R	#3, 4, 5 + 6 Cys (1 unusual disulfide*)	No
Q8WR49	Nematoda	Meja	*Meloidogyne javanica*	K252R	#3, 4, 5 + 6 Cys (1 unusual disulfide*)	No
Fungi						
A0A1Y3NI05	Fungi	Pysp	*Piromyces sp.*		#2, 3, 4, 5+2 Cys (1 unusual disulfide*)	CBM1
A0A1B7XSU6	Fungi	Cohi	*Colletotrichum higginsianum*		#2, 3, 4 ,5 +5 Cys (2 unusual disulfides*)	CBM1
A0A0N0V4Y2	Fungi	Fula	*Fusarium langsethiae*	K252T, R255Q	only #2 and 4 +1 Cys	N-terminal extension
Oomycota						
A0A2P4XIG7	Oomycota	Phpa	*Phytophthora palmivora var. palmivora*		#2, 3, 4 and 5	No
A0A2D4BN86	Oomycota	Pyin	*Pythium insidiosum*		#2, 3, 4 and 5	C-terminal extension
XP_024585360.1	Oomycota	Plha	*Plasmopara halstedii*		#2, 3, 4 and 5	No
XP_024581403.1	Oomycota	Plha	*Plasmopara halstedii*		#2, 3, 4 and 5	No
Fungi						
A0A286UX89	Fungi	Pyno	*Pyrrhoderma noxium*		#2, 3, 4 and 5	two C-terminal domains
A0A166MUN7	Fungi	Coin	*Colletotrichum incanum*		#2, 3, 4, 5 + 4 Cys (2 unusual disulfides*)	CBM1 & DE-rich

G2WR80	Fungi	Veda	*Verticillium dahliae*		#2, 3, 4, 5 + 4 Cys (2 unusual disulfides*)	DEK-rich
A0A0C3QVC1	Fungi	Tuca	*Tulasnella calospora*		#2, 3, 4, 5 + 4 Cys (2 unusual disulfides*)	CBM1
A0A1G4BBA2	Fungi	Coor	*Colletotrichum orchidophilum*		#2, 3, 4, 5 + 2 Cys (1 unusual disulfide*)	C-terminal PBP domain
A0A163KWW1	Fungi	Dira	*Didymella rabiei*		#2, 3, 4 and 5	C-terminal domain
N4VGC5	Fungi	Coor	*Colletotrichum orbiculare*		#2, 3, 4 and 5	D-rich
A0A0J9VDT7	Fungi	Fuox	*Fusarium oxysporum f. sp. lycopersici*		#2, 3, 4 and 5	C-terminal DEK-rich
K1WX96	Fungi	Mabr	*Marssonina brunnea f. sp. multigermtubi*	K252T	#2, 3, 4 and 5	DEK-rich

* - The occurrence of disulfide bonds equivalent to #1, 2, 3, 4 and 5 of Pel3 was verified by structure-based sequence alignments using ESPript server and by protein structure modelling using Swiss Model server (57,58).

Table S3. Heuristic PDB search performed with the Dali server (33) using monomer B of PDB 4U49 as query structure. The first 44 selected PDB entries are shown.

No	PDB-Chain	Z	rmsd	lali	nres	%id	PDB Description
1	4U49-B	21.0	0.1	89	318	100	PECTATE LYASE
2	4U4B-A	18.2	1.0	89	326	100	PECTATE LYASE
3	4U49-A	18.1	1.0	85	311	100	PECTATE LYASE
4	3B8Y-A	17.9	0.7	87	304	48	ENDO-PECTATE LYASE
5	3B4N-A	17.5	1.0	88	314	49	ENDO-PECTATE LYASE
6	3B4N-B	17.3	1.1	88	314	49	ENDO-PECTATE LYASE
7	3B8Y-B	16.6	0.7	82	294	50	ENDO-PECTATE LYASE
8	3MPC-A	12.5	1.7	84	96	25	FN3-LIKE PROTEIN
9	5E53-B	11.7	2.1	85	296	9	CONTACTIN-1
10	4Q58-D	11.6	1.9	84	194	11	PLECTIN
11	1QG3-B	11.5	1.9	84	193	11	PROTEIN (INTEGRIN BETA-4 SUBUNIT)
12	5E53-A	11.5	2.1	85	288	9	CONTACTIN-1
13	1CFB-A	11.4	1.8	82	205	15	DROSOPHILA NEUROGLIAN
14	3F7P-C	11.4	2.0	84	200	11	PLECTIN-1
15	4UI2-A	11.4	1.8	86	201	10	NEOGENIN
16	3F7P-D	11.3	2.0	84	196	11	PLECTIN-1
17	5E4S-A	11.3	1.9	83	301	8	CONTACTIN-4
18	3F7Q-B	11.3	2.0	84	214	11	INTEGRIN BETA-4
19	1QG3-A	11.3	2.0	84	195	11	PROTEIN (INTEGRIN BETA-4 SUBUNIT)
20	3F7Q-A	11.3	2.0	84	214	11	INTEGRIN BETA-4
21	5E53-D	11.3	2.0	84	301	10	CONTACTIN-1
22	3F7P-E	11.2	2.0	84	212	11	PLECTIN-1
23	4N68-A	11.2	2.0	84	99	10	CONTACTIN-5
24	4BQ7-B	11.2	1.9	86	202	12	NEOGENIN
25	4BQ8-A	11.2	1.8	86	195	10	NEOGENIN
26	3F7R-A	11.1	2.1	84	214	11	INTEGRIN BETA-4
27	4BQB-A	11.1	1.9	86	195	10	NEOGENIN
28	4BQB-B	11.1	1.9	86	195	10	NEOGENIN
29	2GEE-A	11.1	2.1	83	188	12	HYPOTHETICAL PROTEIN
30	4BQB-C	11.1	1.9	86	199	10	NEOGENIN
31	4BQ7-A	11.1	1.9	86	202	10	NEOGENIN
32	4BQ6-A	11.0	1.9	86	205	10	NEOGENIN
33	4BQ6-B	11.0	1.9	86	205	10	NEOGENIN
34	4YFD-A	11.0	1.9	84	491	12	INTERLEUKIN-1 RECEPTOR ACCESSORY PROTEIN
35	3P4L-A	11.0	1.9	86	198	10	NEOGENIN
36	4YFG-B	11.0	1.9	85	481	12	RECEPTOR-TYPE TYROSINE-PROTEIN PHOSPHATASE DELTA
37	1V5J-A	10.9	1.9	85	108	13	KIAA1355 PROTEIN
38	4BQ9-B	10.9	2.0	86	187	12	NEOGENIN
39	4BQB-D	10.9	1.9	86	203	10	NEOGENIN
40	4BQC-B	10.9	2.0	86	176	10	NEOGENIN
41	4YFG-A	10.9	1.9	84	481	12	RECEPTOR-TYPE TYROSINE-PROTEIN PHOSPHATASE DELTA
42	5E53-C	10.9	2.1	84	292	10	CONTACTIN-1
43	6MFA-A	10.9	2.0	82	363	11	FIBRONECTIN
44	3T1W-A	10.8	2.1	84	368	14	FOUR-DOMAIN FIBRONECTIN FRAGMENT

Table S4. Plasmids used in this study

Plasmid	Genotype/phenotype	Reference
pBS	Bluescript KS+, ColE1, Ap^R	Stratagene
pBS-PLI	pBS carrying *pelI* of *D. dadantii* 3937	(20)
pBS-PL3	pBS carrying *pel3* of *P. carotovorum* 71	(20)
pBS-h16	pBS carrying a chimeric *pelI-pel3* encoding residues M1-D91 from Pel3 and V90-L344 from PelI	This work
pBS-h16-2	pBS-h16 carrying additional substitutions in the loop 2 of Fn3 domain: A43E, G44S, N45K	This work
pBS-h16-3	pBS-h16 carrying additional substitutions in the loop 3 of Fn3 domain: S54G, S56T, A58N, Q59P, A60D, G61L, S62R	This work
pBS-h16-4	pBS-h16 carrying additional substitutions in the loop 4 of Fn3 domain: N69D, S70A, S71E, D72T	This work
pBS-h16-5	pBS-h16 carrying additional substitutions in the loop 5 of Fn3 domain: L78A, T79D, A80T, P82S, Q83G, S84L, D85N	This work
pBS-h16-3,2	pBS-h16-3 carrying additional substitutions in the loop 2 of Fn3 domain: A43E, G44S, N45K	This work
pBS-h16-3,4	pBS-h16-3 carrying additional substitutions in the loop 4 of Fn3 domain: N69D, S70A, S71E, D72T	This work
pBS-h16-3,5	pBS-h16-3 carrying additional substitutions in the loop 5 of Fn3 domain: L78A, T79D, A80T, P82S, Q83G, S84L, D85N	This work
pBS-h18	pBS carrying a chimeric *pelI-pel3* encoding residues M1-A128 from Pel3 and T126-L344 from PelI	This work
pBS-h18-C	pBS-h18 carrying additional substitutions in the loop T1.8 of catalytic domain: N316K, E319V, K322Q, K324S, S325T, D326E	This work
pBS-h18-B,C	pBS-h18-C carrying additional substitutions in the loop T3.4 of catalytic domain: T217A, T218K, N219D, P221Y	This work
pBS-h27	pBS carrying a chimeric *pelI-pel3* with several substitutions shown on Fig. 3B and S9	This work
pBS-h33	pBS carrying a chimeric *pelI-pel3* with several substitutions shown on Fig. 3B and S9	This work
pBS-h35	pBS carrying a chimeric *pelI-pel3* with several substitutions shown on Fig. 3B and S9	This work
pBS-PLI_70/240C	pBS-PLI carrying T70C and N240C substitutions	This work
pBS-PLIstp	pBS-PLI carrying amber stop codon in the place of P107	This work

Table S5. Primers employed in the study

Primer	Nucleotide sequence (5'-3')[b]	Generated mutation
Pel3_PS [a]	cttttcaggctcaggctg**c**t**c**a**g**accatgctgatgctgctg	D22A, D23Q
Pel3_lp2asl [a]	ttaagctggtctaccgatg**a**a**a**gcaa**a**gttgttcgccaggatgtg	A43E, G44S, N45K
Pel3_lp3asl [a]	gttcgccaggatgtgtatcgc**g**gcacca**c**tagt**aa**tc**c**gg**atct**c**c**gcgaaaaaatcgcagagctc	S54G, S56T, A58N, Q59P, A60D, G61L, S62R
Pel3_lp4asl_sh [a]	gcgaaaaaatcgcagagctc**g**at**g**cc**gagac**cagaacctttaccgatttaaccg	N69D, S70A, S71E, D72T
Pel3_lp5asl [a]	ccagcgacagaacctttaccgat**gc**a**ga**c**a**ccaat**t**ca**gg**gt**t**a**a**actattggtattgggtggatacc	L78A, T79D, A80T, P82S, Q83G, S84L, D85N
Pel3_lp6asl [a]	gggtggataccgttagcg**ag**aat**c**a**ggc**c**cagg**tg**gt**atctaatgctgcctcaacagc	G95E, N97Q, S98A, V99Q, L100V, K101V
Pel3_T3.1asl [a]	gtggtattacgctgggt**ac**gagctgt**cc**c**aa**tgacagtgataaacagcc	L144T, S147P, G148N
Pel3_T3.4asl [a]	gtcggcggtgtggcacataac**g**cca**aag**atggt**ta**tggcggcaaaccggacaaagtg	T217A, T218K, N219D, P221Y
Pel3_T1.8asl_Ct [a]	cggtgtagaaaaaggg**c**aaggaa**gca**ccga**g**aaatacggagagttctgg	K322Q, K324S, S325T, D326E
Pel3_T1.8asl_Nt [a]	ggtatgtgaagagtttaa**a**ggtgtagt**a**aaaggg**c**aaggaagcaccg	N316K, E319V, K322Q
Pel3_Ehe [a]	ccagaatgtaaagccgg**c**gc**c**gtaattaaagataaaaccg	*Ehe*I site covering G127 and A128
Pel3_Aat [a]	ctattggtattgggtgga**cgt**cgttagcggtaataatagcg	*Aat*II site, T92V
PelI_T70C [a]	cgccgtgctggacgcggaa**tg**ccgtacctttaaagatgccgac	T70C
PelI_N240C [a]	gcaccaccgtggtgaagggc**tg**cttcaccctgaccggtgaacac	N240C
PelI_NtSt [a]	gacggttatggcggcaaa**tga**gacaaagtgctgcagcac	P222tga
Pel3_EcoRV [a]	cttttcaggctcaggctgat**at**caccatgctgatgctgctg	*Eco*RV site, D23I

[a] For each primers used in site directed mutagenesis, another primer with reverse complementary sequence was used (not shown).

[b] Mutated or introduced bases are in bold and underlined.

Supporting Experimental procedures

Plasmid constructions

To generate pBS-h16 plasmid carrying chimeric *pelI-pel3* gene encoding the residues M1-D91 from Pel3 and V90-L344 from PelI, an *Aat*II site was introduced into the *pel3* gene in the zone coding for T92 using Pel3_Aat primer pair (Table S5). Next, the 5' region of *pel3* was fused to the 3' region of *pelI* through the *Aat*II site. To generate pBS-h18 plasmid carrying chimeric *pelI-pel3* gene encoding the residues M1-A128 from Pel3 and T126-L344 from PelI, an *Ehe*I site was introduced into *pel3* in the zone coding for G127 and A128 using Pel3_Ehe primer pair (Table S5). Next, the 5' region of *pel3* was fused to the 3' region of *pelI* through the *Ehe*I site. Additional substitutions in h16 and h18 derivatives was introduced by site-directed mutagenesis with the PrimeSTAR Max DNA Polymerase (TaKaRa) using the appropriate primer pairs listed in Table S5. To generate pBS-h33, the multiple substitutions shown on Fig. S9, were sequentially introduced by site-directed mutagenesis using the appropriate primer pairs listed on Table S5. To generate pBS-h27, an *Aat*II site was introduced into the *h16-3,5* gene in the zone coding for T92 using Pel3_Aat primer pair (Table S5). Next, the 5' region of *h16-3,*5 was fused to the 3' region of *h18_B,C* through the *Aat*II site.
